# Efficacy and Safety of Transcutaneous Electrical Nerve Stimulation in Patients Undergoing Inguinal Hernia Repair: A Systematic Review and Meta-analysis

**DOI:** 10.31662/jmaj.2023-0056

**Published:** 2023-09-13

**Authors:** Jun Watanabe, Naoto Izumi, Fuyumi Kobayashi, Atsushi Miki, Naohiro Sata

**Affiliations:** 1 Department of Surgery, Division of Gastroenterological, General and Transplant Surgery, Jichi Medical University, Shimotsuke, Japan; 2 Division of Community and Family Medicine, Jichi Medical University, Shimotsuke, Japan

**Keywords:** inguinal hernia repair, meta-analysis, pain, systematic review, transcutaneous electrical nerve stimulation

## Abstract

**Background:**

Postoperative pain is a major cause of delayed recovery following inguinal hernia repair. Transcutaneous electrical nerve stimulation (TENS) is a simple, low-cost method of noninvasive analgesia. This study aimed to assess the efficacy and safety of TENS for pain management following inguinal hernia repair.

**Methods:**

We searched nine electronic databases and trial registries to identify randomized controlled trials (RCTs). The primary outcomes were postoperative pain and the use of rescue analgesics. The Risk of Bias 2 tool was used to evaluate the risk of bias in the included trials. The certainty of the evidence was assessed using Grading of Recommendations, Assessment, Development, and Evaluations (GRADE). Subgroup analyses were conducted based on the anesthesia type or TENS dose and frequency. This study is registered with PROSPERO (CRD42022353932).

**Results:**

Eleven RCTs, with a total of 559 patients, were included. The overall risk of bias was concerning due to the lack of information about concealment or published protocols. TENS may reduce pain on postoperative day (POD) 0 (standardized mean difference [SMD], −2.14; 95% confidence interval [CI], −3.54 to −0.73; moderate certainty of the evidence), POD 1 (SMD, −1.22; 95% CI, −1.92 to −0.52; moderate certainty of the evidence), and POD 2 (SMD, −0.97; 95% CI, −2.04 to 0.10; low certainty of the evidence). According to the subgroup analyses, postoperative pain was reduced, particularly with local anesthesia or repetitive and frequent TENS (*P* < 0.05). TENS may result in little-to-no difference in rescue analgesic use (risk ratio, 0.75; 95% CI, 0.47-1.18; low certainty of the evidence). No serious adverse events occurred (moderate certainty of the evidence).

**Conclusions:**

TENS may reduce pain in patients who have undergone inguinal hernia repair. Further trials are warranted to confirm our findings.

## Introduction

Inguinal hernia repair surgery is one of the most frequent general surgeries and is performed in more than 20 million patients annually worldwide, accounting for approximately one-third of all surgical interventions ^(1)^. Recovery time following hernia repair surgery is a major socioeconomic issue due to residual pain that interferes with the patient’s daily life and employment; this pain occurs in approximately 6% of patients following hernia repair surgery ^(2)^. Moderate or severe pain after hernia repair surgery is common and peaks on postoperative day (POD) 1 ^(3)^. It is hypothesized that active analgesia can avoid central sensitization from incisional and inflammatory stimuli during the early postoperative period ^(4)^. As a result, the risk of developing chronic pain may be reduced ^(5)^. Nonsteroidal anti-inflammatory drugs and opioids are often used for postoperative pain; however, opioids for postoperative pain exert many undesirable side effects, including nausea, vomiting, urinary retention, sedation, respiratory depression, drug dependence, dizziness, and dry month ^(6)^. Therefore, it is important to reduce pain and analgesic consumption after hernia repair surgery to achieve rapid recovery and enable return to normal activities ^(7), (8)^.

Transcutaneous electrical nerve stimulation (TENS) is a noninvasive, transcutaneous method of electrical stimulation that produces analgesia ^(9)^ and is also portable and inexpensive; furthermore, the device used produces a mild pulsed current that is delivered across the skin surface to stimulate peripheral nerves *via* electrode pads ^(10)^. Conventional TENS is applied at high frequencies (50-130 Hz), at a low intensity (comfortable and painless), and with short pulse durations (50-200 μs) ^(11)^. It selectively activates nonpainful, low-threshold afferent nerve fibers (Aβ fibers) in the skin, thus inhibiting the transmission of nociceptive information at the level of the spinal cord to provide analgesia ^(12)^. A previous Cochrane review evaluated the analgesic effects of TENS on acute pain in adults; however, these effects could not be confirmed due to the clinical heterogeneity caused by the integration of various acute pain conditions ^(13)^. No comprehensive systematic reviews of TENS have focused on inguinal hernia repair.

Therefore, we aimed to assess the efficacy and safety of TENS in patients who have undergone inguinal hernia repair.

## Materials and Methods

We followed the Preferred Reporting Items for Systematic Reviews and Meta-analysis (PRISMA) 2020 ^(14)^. This study is registered with PROSPERO (CRD42022353932), and the protocol is registered in OSF (https://osf.io/6qctw/).

Institutional review board approval was not required for this study as all data were retrieved from published articles.

### Selection criteria

We included individual randomized controlled trials (RCTs) and excluded crossover, cluster, and nonrandomized trials. Furthermore, we included trials that compared adults older than 18 years who underwent hernia repair surgery. We excluded participants who had not undergone inguinal hernia repair when they were included in the study population and those with electronic implants, such as cardiac pacemakers and implantable cardioverter defibrillators. We included trials that compared TENS with placebo or usual care. In addition, we included the following cointerventions if they were not part of the randomized treatment: antibiotics, analgesics, and antiemetics.

The primary outcomes were pain on POD 1 and the use of rescue analgesics, whereas the secondary outcomes were pain on PODs 0 and 2, quality of life, and all adverse events. Pain following hernia repair surgery was defined using the mean visual analog scale (VAS) or numeric rating scale (NRS) regardless of whether the pain occurred at rest or when performing activity ^(8)^. When pain was measured several times, the last score was used. When pain at rest and pain during activities were measured, the former was used. The proportion of patients requiring rescue analgesics was defined as the number of patients requiring rescue analgesics divided by the total number of patients during postoperative hospitalization. Quality of life was evaluated using the Short Form (SF)-36 total score.

### Study selection, data extraction, and risk of bias

We performed a literature search to find all published and unpublished RCTs regardless of language on the following electronic databases: Cochrane Central Register of Controlled Trials (CENTRAL), MEDLINE *via* PubMed (1966 to present), EMBASE (1988 to present), CINAHL (1982 to present), AMED (1985 to present), PsycINFO (1806 to present), and Web of Science (1956 to present) (Supplementary 1). We checked the reference lists of all eligible trials to collect additional references. We further performed a search on Clinical Trials.gov and the World Health Organization International Clinical Trials Platform Search Portal (ICTRP) (Supplementary 2). We checked the references of the guidelines ^(1), (15)^. Furthermore, we contacted the authors of the identified trials and asked them to confirm any missing data.

Two review authors (JW and NI) independently screened the titles and abstracts for inclusion, screened the full text, and extracted the study characteristics and outcomes. We resolved any disagreement through discussion or consultation with a third author (FK or AM). Two of the three review authors (JW and FK or NI) independently evaluated the risk of bias using the Risk of Bias 2 tool ^(16)^. We resolved any disagreement through discussion or consultation with a third review author (AM).

A table containing summary of findings was created for the outcomes based on the Cochrane handbook ^(17)^. We evaluated the quality of evidence based on the Grading of Recommendations, Assessment, Development, and Evaluation (GRADE) approach for each table ^(18)^.

### Data synthesis

We analyzed the relative risk ratios and 95% confidence intervals (CIs) of the rescue analgesics and the continuous data of pain using the standardized mean difference (SMD) and quality of life using the mean difference. We decided to use the SMD for pain as pain was measured using different scales, such as VAS and NRS, according to the Cochrane handbook ^(17)^. We summarized adverse events based on the definition in the original article; however, we did not conduct meta-analyses. We asked the original authors regarding any unreported data. We conducted an intention-to-treat analysis of all dichotomous data when possible. In accordance with the recommendation of the Cochrane handbook, we did not impute missing continuous data ^(17)^. We conducted a meta-analysis of the available data presented in the original study using the Review Manager software (RevMan 5.4.2). We used a random-effects model by default.

We evaluated the statistical heterogeneity by visual inspection of the forest plots and calculation of the *I^2^* statistics (*I^2^* values of 0%-40%, might not be important; 30%-60%, may represent moderate heterogeneity; 50%-90%, may represent substantial heterogeneity; and 75%-100%, considerable heterogeneity) based on the Cochrane handbook ^(17)^. We assessed the cause of heterogeneity when it was substantial (*I^2^* > 50%). The Cochrane χ^2^ test (Q-test) was performed for *I^2^* statistics, and *P* < 0.10 was considered statistically significant.

We performed an extensive literature search on ClinicalTrials.gov and ICTRP for unpublished trials. When we pooled more than 10 trials, we created and examined a funnel plot to explore possible publication bias ^(17)^. We used the Egger test to determine the statistical significance of the reporting bias. *P* < 0.05 was considered to indicate statistical significance for reporting bias.

### Additional analyses

We conducted subgroup analyses of the surgical approach (anterior approach vs. laparoscopy) and TENS type (acupuncture-like-TENS [visible phasic muscle contractions] vs. conventional TENS [no visible muscle contraction]) ^(13)^. We modified our protocol to add subgroup analyses of the anesthesia type and TENS dose and frequency.

We conducted a sensitivity analysis to evaluate the robustness of our conclusions. Studies using imputed statistics and those with incomplete data were excluded. However, we could not conduct a sensitivity analysis of the excluded studies using imputed statistics as there were none.

## Results

**Figure 1.**
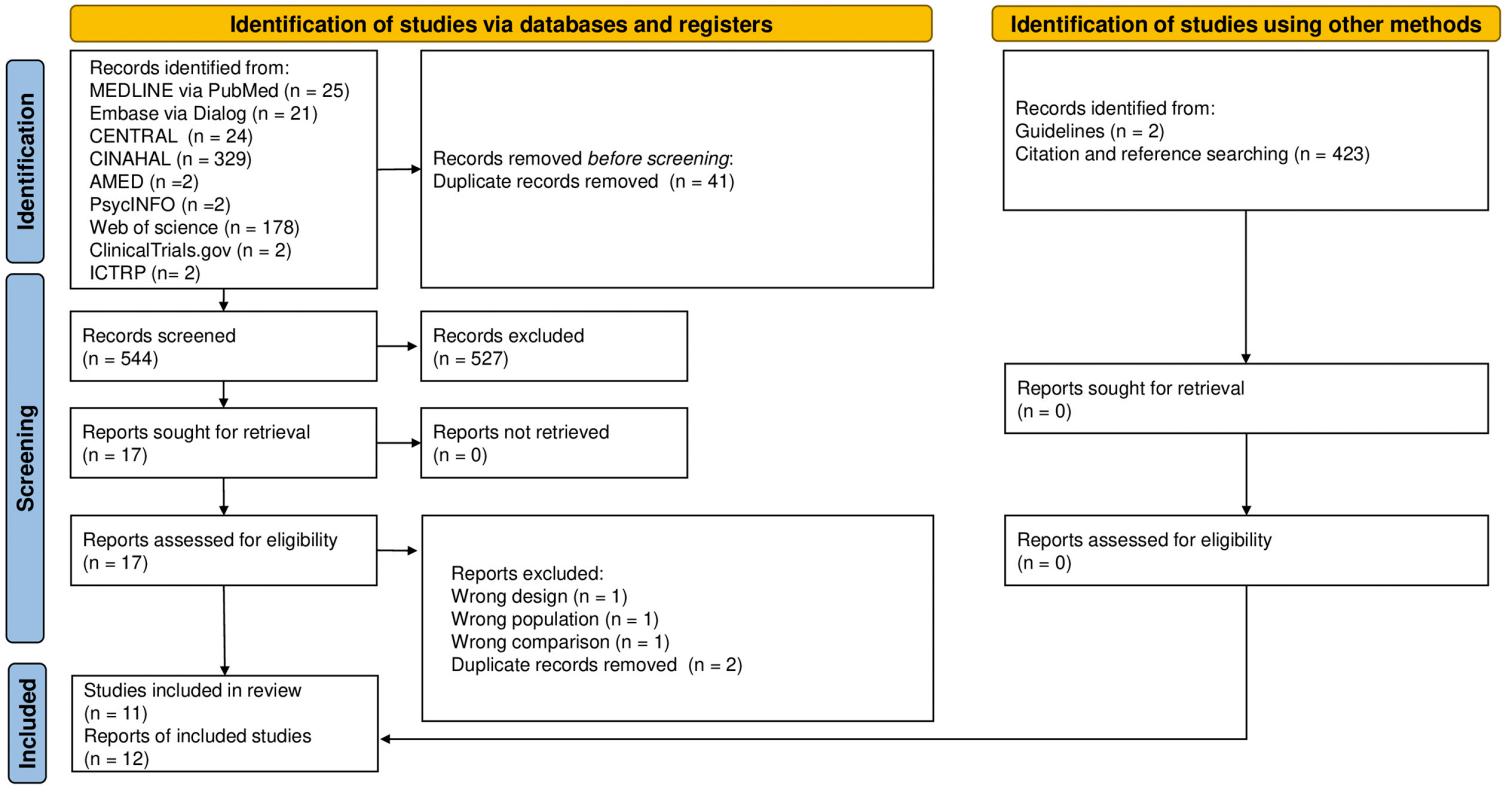
Flow of the study selection process.

We identified 585 citations, including 544 unique reports. Of these, we screened 18 full-text articles after excluding 527 reports based on the titles and abstracts. After the full-text screening, three articles were excluded, one because of its study design, the other because of its population (IRCT138706101061N2), and another one because of its comparisons. We included 11 RCTs (12 reports) involving 559 patients (Figure 1) ^(19), (20), (21), (22), (23), (24), (25), (26), (27), (28), (29), (30)^.

The eligibility criteria are presented in Table 1. Ten trials used the anterior approach and one used laparoscopy for inguinal hernia. Nine trials used conventional TENS approaches, and two used acupuncture-like TENS. Two trials used one TENS dose, two used two doses, and six used multiple doses. Nine trials used high-frequency TENS, one opted for low-frequency TENS, and one had no reported frequency. Ten trials used the VAS, and one trial used the NRS. There were no patients with epidural anesthesia in the included RCTs. Table 2 and Supplementary 3 present the risk of bias of the eligibility studies of pain. In 11 studies, the overall risk of bias was concerning due to the lack of information about concealment or published protocols.

**Table 1.** Summary of the Characteristics of the Eligibility Studies.

Authors [ref no.]	Year	Country	Subject no. (intervention/control)	Age (years) (intervention/control)	Surgical approach	Intervention (TENS)	Dose of TENS	Frequency (Hz)	Control	Pain scale	Postoperative analgesic regimen	Anesthesia (local/spinal/general)	Follow-up (day)
Gilbert ^(19)^	1986	UK	40 (20/20)	50/56	Anterior approach	Conventional	Single	70	Sham	VAS	Nothing	0/40/0	3
Smedley ^(20)^	1988	UK	62 (34/28)	57/55	Anterior approach	Conventional	Single	70	Sham	VAS	Nothing	0/0/62	2
DeSantana ^(21)^	2008	USA	40 (20/20)	49/42	Anterior approach	Conventional	Twice	100	Sham	NRS	Dipyrone 1 g	0/40/0	1
Ahmed ^(22)^	2010	Egypt	60 (30/30)	36/34	Anterior approach	Conventional	Repetitive	100	Sham	VAS	Paracetamol 500 mg	67/0/0	5
Dias ^(23)^	2010	Brazil	33 (16/17)	47/43	Anterior approach	AL-TENS	Twice	240	Sham	VAS	Nothing	33/0/0	14
Dalamagka ^(24)^	2015	Greece	36 (18/18)	54/53	Anterior approach	AL-TENS	Repetitive	2	Sham	VAS	Pethidine 15 mg	0/0/36	2
Eidy ^(25)^	2016	Iran	66 (33/33)	34/33	Anterior approach	Conventional	Single	NR	Sham	VAS	PCA (pethidine 4 mg/hour)	0/0/66	1
Gorganchian ^(26)^	2016	Argentina	24	NR	Anterior approach	Conventional	Repetitive	100	Sham	VAS	Tramadol 50 mg	NR	1
Yilmaz ^(27)^	2019	Turkey	52 (26/26)	45/50	Anterior approach	Conventional	Repetitive	100	Sham	VAS	Diclofenac 75 mg	NR	1
Parseliunas ^(28), (30)^	2021	Lithuania	80 (40/40)	62/61	Anterior approach	Conventional	Repetitive	100	Sham	VAS	Ketoprofen 100 mg	0/80/0	2
Szmit ^(29)^	2021	Poland	48 (24/24)	64/61	Laparoscopy	Conventional	Repetitive	100	Sham	VAS	PCA (morphine 1 mg/time)	0/0/48	1

AL-TENS, acupuncture-like transcutaneous electrical nerve stimulation; Intra, intraoperative; NR, not reported; NRS, numeric rating scale; PCA, patient-controlled analgesia; Pre, preoperative; Post, postoperative; VAS, visual analog scale.

**Table 2.** Risk of Bias for the Eligibility Studies for Patients’ Pains.

Authors [ref no.]	Risk of bias 2 tool assessment					
	Bias arising from the randomization process	Bias due to deviations from the intended interventions	Bias due to missing outcome data	Bias in the measurement of the outcome	Bias in the selection of the reported results	Overall risk of bias
Gilbert ^(19)^	Some concerns	Low	Low	Some concerns	Some concerns	Some concerns
Smedley ^(20)^	Some concerns	Low	Low	Some concerns	Some concerns	Some concerns
DeSantana ^(21)^	Low	Low	Low	Some concerns	Some concerns	Some concerns
Ahmed ^(22)^	Low	Low	Some concerns	Some concerns	Low	Some concerns
Dias ^(23)^	Low	Low	Low	Low	Some concerns	Some concerns
Dalamagka ^(24)^	Low	Low	Low	Low	Some concerns	Some concerns
Eidy ^(25)^	Low	Low	Low	Some concerns	Low	Some concerns
Gorganchian ^(26)^	Some concerns	Low	Low	Some concerns	Some concerns	Some concerns
Yilmaz ^(27)^	Some concerns	Some concerns	Some concerns	Some concerns	Some concerns	Some concerns
Parseliunas ^(28), (30)^	Low	Low	Low	Some concerns	Low	Some concerns
Szmit ^(29)^	Low	Low	Low	Some concerns	Low	Some concerns

The risk of bias using Risk of Bias 2 tool; Low: the risk of bias was low. Some concerns: the risk of bias was some concerns. High: the risk of bias was high.

### Outcomes

The evidence related to each outcome is summarized in Table 3.

**Table 3.** Summary of Findings.

Efficacy and safety of transcutaneous electrical nerve stimulation in patients undergoing inguinal hernia repair						
Patient or Population: Adults, Setting: Inguinal hernia repair, Intervention: TENS, Comparison: Sham						
Outcomes	Anticipated Absolute Effects * (95% CI)		Relative Effect (95% CI)	Patient Number (Studies)	Certainty of the Evidence (GRADE)	Comments
	Risk with control	Risk with TENS				
Patients’ pain (POD1)	-	SMD −1.22 (−1.92 to −0.52)	-	559 (11 RCTs)	Moderate ^a,b,c^	TENS may result in a large reduction in pain at POD 1.
Rescue analgesic use	569 per 1000	427 per 1000 (267 to 672)	RR 0.75 (0.47 to 1.18)	252 (5 RCTs)	Low ^a,b^	TENS may result in little to no difference in rescue analgesic use.
Patients’ pain (POD 0)	-	SMD −2.14 (−3.54 to −0.73)	-	307 (6 RCTs)	Moderate ^a,b,c^	TENS likely result in a large reduction in pain at POD 0.
Patients’ pain (POD 2)	-	SMD −0.97 (−2.04 to 0.10)	-	275 (5 RCTs)	Low ^a,b^	TENS may slightly reduce pain at POD 2.
Quality of life Assessed with SF-36	The mean score was 53.02	MD 2.58 (−3.61 to 8.77)	-	73 (1 RCT)	Very low ^b,d^	The evidence is very uncertain about the effect of TENS on the quality of life
Adverse events	In one patient in the TENS group and two patients in the sham group, skin irritation (redness and itching) was observed.			242 (3 RCTs)	Moderate ^a^	No serious adverse events were observed.

CI, confidence interval; MD, mean difference; POD, postoperative day; RR, risk ratio; SMD, standard mean difference; TENS, transcutaneous electrical nerve stimulation. * The risk in the intervention group (and its 95% CI) is based on the assumed risk in the comparison group and the relative effect of the intervention (and its 95% CI). GRADE Working Group grades of evidence; High certainty: We are very confident that the true effect lies close to that of the estimated effect. Moderate certainty: We are moderately confident in the estimated effect. The true effect is likely to be close to the estimated effect, but there is a possibility that it is significantly different. Low certainty: Our confidence in the estimated effect is limited: The true effect may be significantly different from the estimated effect. Very low certainty: We have very little confidence in the estimated effect. The true effect is likely to be significantly different from the estimated effect. a Downgraded one point because of inconsistency due to substantial heterogeneity. b Downgraded one point because of imprecision due to the small sample size. c. Upgraded one point because of the large effect size. d. Downgraded two points because of imprecision due to only one study.

### Primary outcomes

#### Postoperative pain on POD 1

**Figure 2.**
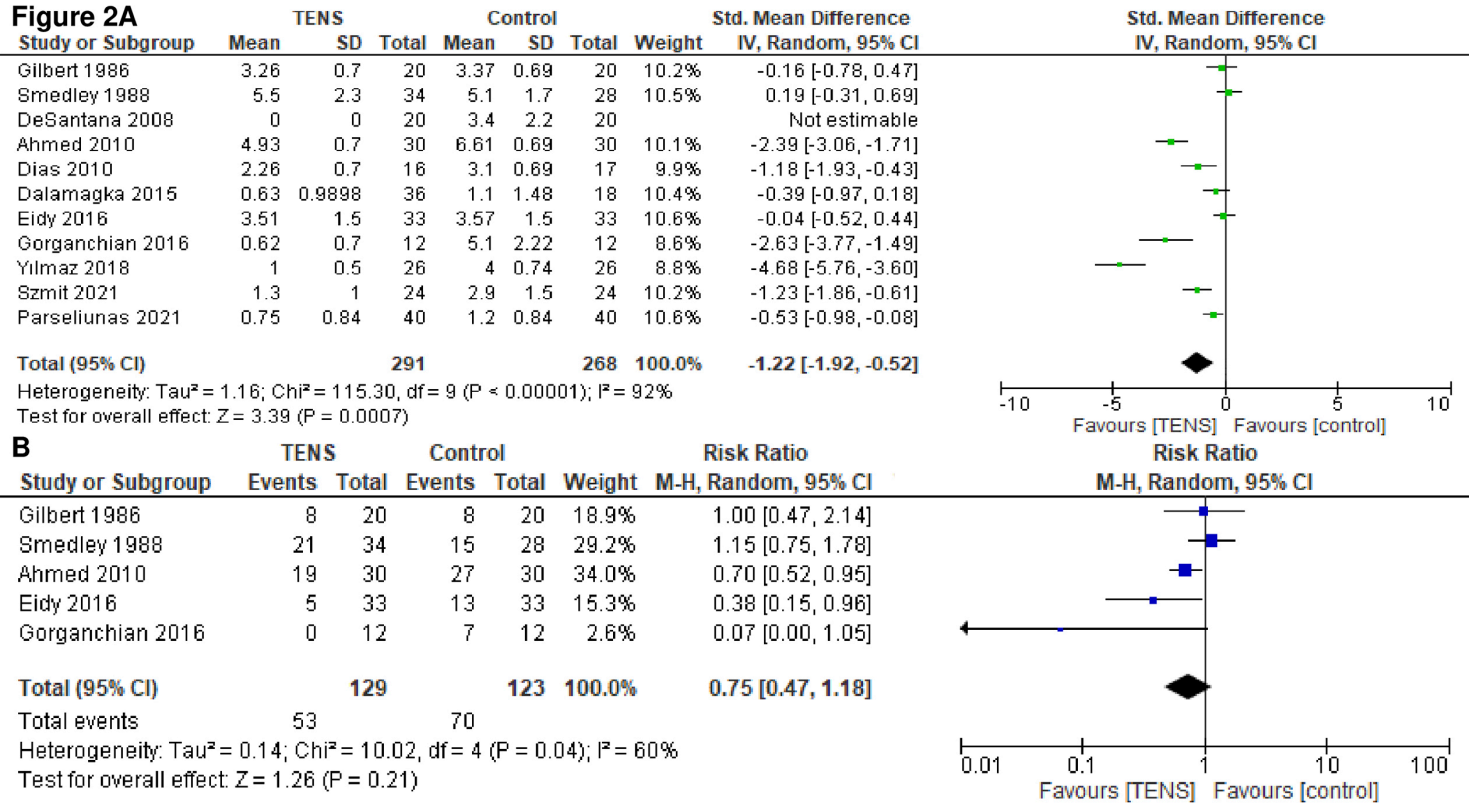
Forest plot A) pain on postoperative day 1; B) proportion of patients who received rescue analgesics.

A total of 11 studies involving 559 patients reported pain on POD 1 ^(19), (20), (21), (22), (23), (24), (25), (26), (27), (28), (29), (30)^. Among them, 10 used the VAS, whereas 1 used the NRS. TENS likely resulted in a large reduction of postoperative pain on POD 1 (SMD, −1.22; 95% CI, −1.92 to −0.52;* I^2^* = 92%; moderate certainty of the evidence) (Figure 2A). In the subgroup analyses (Supplementary Figure 1), no differences were observed in the surgical approach or TENS type (*P* > 0.05); however, differences were found in the anesthesia type and TENS dose and frequency (*P* < 0.05). For patients who had undergone local anesthesia, TENS reduced postoperative pain on POD 1 (SMD, −2.68; 95% CI, −4.00 to −1.35; *I^2^* = 89%) but it did not reduce the postoperative pain of patients who underwent general anesthesia (SMD, −0.46; 95% CI, −1.27 to −0.34;* I^2^* = 84%) or spinal anesthesia (SMD, −0.27; 95% CI, −0.58 to 0.05; *I^2^* = 14%) on POD 1. Repetitive TENS reduced postoperative pain on POD 1 (SMD, −1.90; 95% CI, −2.96 to −0.84; *I^2^* = 93%); however, single TENS did not (SMD, 0.02; 95% CI, −2.96 to −0.84; *I^2^* = 93%). High-frequency TENS reduced postoperative pain on POD 1 (SMD, −1.50; 95% CI, −2.39 to −0.61; *I^2^* = 93%); however, low TENS did not (SMD, −0.39; 95% CI, −0.97 to −0.18). Funnel plots were visualized as symmetrical, thus indicating minimal publication bias for pain on POD 1 (Egger test = 0.84) (Supplementary Figure 2).

#### Use of rescue analgesics

A total of 5 studies with 252 patients reported the proportion of patients administered rescue analgesics ^(19), (20), (22), (25), (26)^. TENS may result in little to no difference in the proportion of patients requiring rescue analgesics (risk ratio, 0.75; 95% CI, 0.47-1.18; *I^2^* = 60%; low certainty of the evidence) (Figure 2B). In the subgroup analyses (Supplementary Figure 3), no differences were observed in anesthesia type or TENS dose (*P* > 0.05). Subgroup analyses of the surgical approach and TENS type and frequency could not be conducted.

### Secondary outcomes

#### Postoperative pain on POD 0

**Figure 3.**
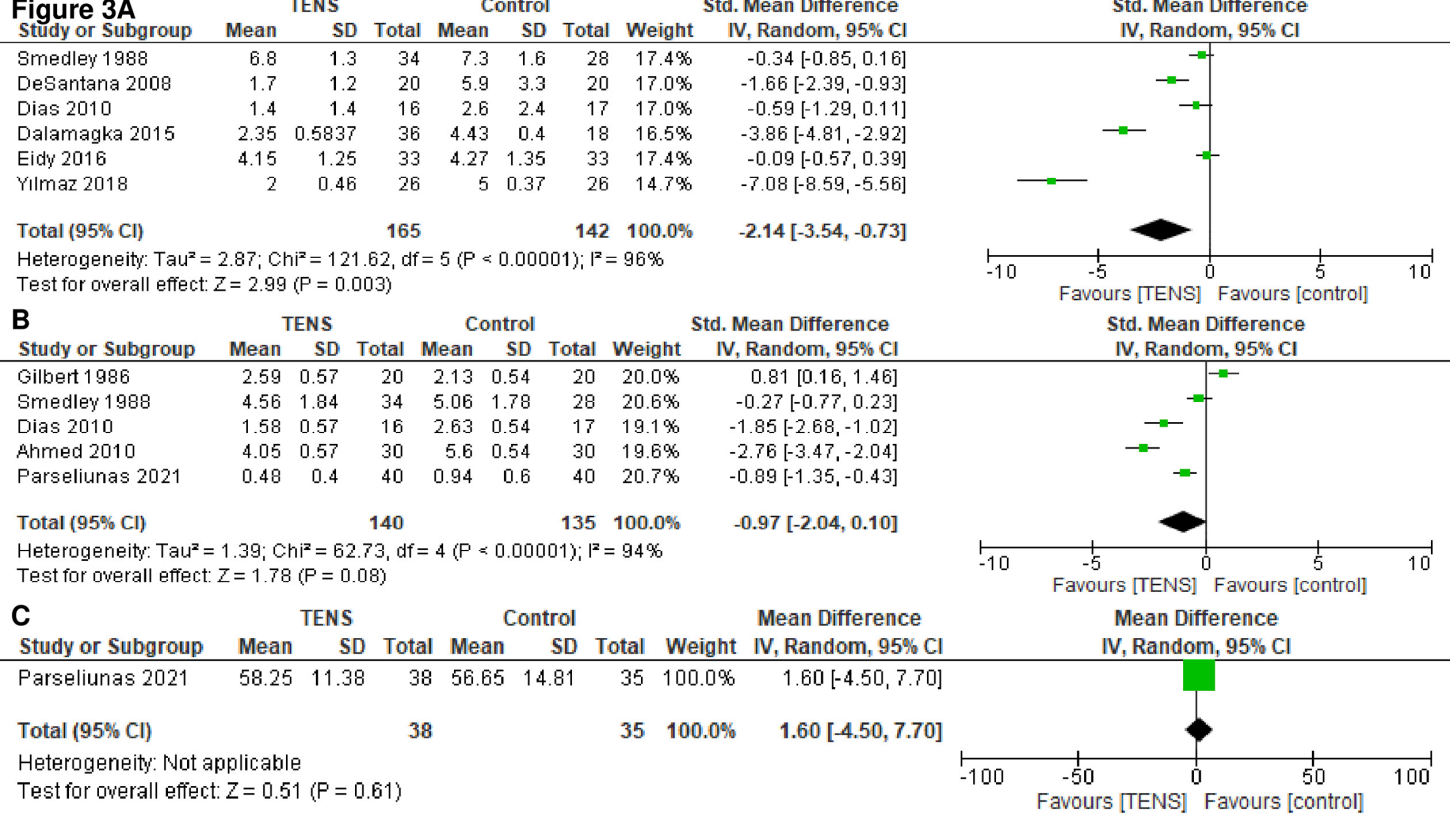
Forest plot A) pain on postoperative day 0; B) pain on postoperative day 2; C) quality of life.

A total of 6 studies with 307 patients reported pain on POD 0 ^(20), (21), (23), (24), (25), (27)^. TENS may result in a large reduction of postoperative pain on POD 0 (SMD, −2.14; 95% CI, −3.54 to −0.73;* I^2^* = 92%; moderate certainty of the evidence) (Figure 3A). In the subgroup analyses (Supplementary Figure 4), no differences were observed in the TENS type and frequency or anesthesia type (*P* > 0.05); however, differences were found in the TENS dose (*P* < 0.05). Subgroup analyses of the surgical approach could not be conducted. Repetitive TENS reduced postoperative pain on POD 0 (SMD, −5.41; 95% CI, −8.56 to −2.26; *I^2^* = 92%), but single TENS administration did not (SMD, −0.21; 95% CI, −0.56 to 0.14; *I^2^* = 0%).

#### Postoperative pain on POD 2

A total of 5 studies with 275 patients reported pain on POD 2 ^(19), (20), (22), (23), (28)^. TENS may reduce postoperative pain on POD 2 (SMD, −0.97; 95% CI, −2.04 to 0.10; *I^2^* = 94%; low certainty of the evidence) (Figure 3B). In the subgroup analyses (Supplementary Figure 5), no differences were observed in TENS type (*P* > 0.05); however, there were differences in the anesthesia type and TENS dose (*P* < 0.05). Subgroup analyses of the surgical approach and TENS frequency could not be conducted. TENS reduced postoperative pain on POD 2 for patients who received local anesthesia (SMD, −2.33; 95% CI, −3.22 to −1.44; *I^2^* = 62%) but not for those who received general anesthesia (SMD, −0.27; 95% CI, −0.77 to 0.23) or spinal anesthesia (SMD, −0.06; 95% CI, −1.73 to 1.61; *I^2^* = 94%). Repetitive TENS may reduce postoperative pain on POD 2 (SMD, −1.80; 95% CI, −3.63 to 0.02; *I^2^* = 95%), but single TENS did not (SMD, 0.25; 95% CI, −0.81 to 1.31; *I^2^* = 85%). The results of the sensitivity analyses were consistent with those of the primary analyses (Supplementary Figure 6).

#### Quality of life

In one study, quality of life was evaluated using the total SF-36 score ^(30)^. The evidence of the effect of TENS on the quality of life is uncertain (mean difference, 2.58; 95% CI, 0.47-1.18; very low certainty of the evidence) (Figure 3C).

#### Adverse events

Three studies reported adverse events ^(21), (23), (29)^. One patient in the TENS group and two in the sham group had skin irritation (redness and itching). No serious adverse events occurred. The certainty of the evidence was moderate.

## Discussion

This review of 11 RCTs that included 559 patients demonstrated that TENS may reduce pain until day 2 following inguinal hernia repair; however, TENS may result in little to no difference in the use of rescue analgesics. Differences were observed in the ability of TENS to reduce pain; such differences were dependent on the anesthesia type or TENS dose and frequency. No serious adverse events were observed. Furthermore, the current evidence indicates that the effect of TENS on the quality of life is uncertain. This is the first systematic review to demonstrate that TENS is useful for relieving pain following inguinal hernia repair.

TENS should be used in clinical practice if desired by patients after inguinal hernia repair, particularly those administered local anesthesia or repetitive and frequent TENS, as it reduces pain for up to POD 2 without increasing adverse events. Previous systematic reviews have demonstrated that TENS reduced the pain intensity of adults with acute pain but that acute pain conditions varied and clinical heterogeneity existed ^(13), (31)^. In addition, the duration of the effect of TENS on acute pain was inconclusive. Previous systematic reviews reported that the efficacy of TENS for specific types of acute pain, such as labor pain ^(32), (33)^ and dysmenorrhea, has not been proven ^(34)^. Our study corroborated the results of these previous reviews and extended them by focusing on patients who underwent inguinal hernia repair and showing that TENS reduced pain on POD 2. According to Cohen’s interpretation, pain reduction following inguinal hernia repair was considered significant at −0.8 or less ^(35)^. It is noteworthy that even though local anesthesia is superior to general anesthesia for pain reduction following inguinal hernia repair ^(36)^, our subgroup analysis revealed that TENS may be more effective for patients who have undergone local anesthesia. Similar to the results of a previous review of TENS for knee osteoarthritis ^(37)^, our subgroup analysis revealed that repetitive TENS was more effective than single TENS. Comparable with the results of a previous review of TENS for acute pain ^(13)^, our subgroup analysis showed that high-frequency TENS was more effective than low-frequency TENS. Although internal guidelines suggest that laparoscopy reduces postoperative pain better than the anterior approach ^(1)^, our subgroup analysis found no significant difference in postoperative pain on POD 1 between the two approaches. Because our review included only one trial with laparoscopy, additional trials are warranted to help elucidate the role of TENS in pain management for patients undergoing laparoscopic procedures and possibly lead to improved postoperative outcomes.

In this review, TENS did not reduce the proportion of patients requiring rescue analgesics. The reason for the absence of differences is unclear; however, it may have been caused by the different postoperative analgesic regimens. Although protocols should predetermine when rescue analgesics is crucial, the outcomes of rescue analgesics use and doses were predetermined by the protocols reported by one of the five studies. In this review, 10 studies used the VAS and 1 used the NRS. In a previous systematic review, the NRS was more sensitive than the VAS for pain assessment ^(38)^. Further studies should use the NRS after determining the timing of rescue analgesic use indicated by the protocol.

In this review, the evidence of the effect of TENS on the quality of life was uncertain as only one included study reported the quality of life. Severe chronic pain following inguinal hernia repair affects the SF-36 score, a valid indicator of overall health, social life, daily activities, and overall quality of life ^(39)^. However, as TENS is designed to improve acute postoperative pain, the Carolinas Comfort Scale allows better assessment of the quality of life and satisfaction of patients who have undergone surgical hernia repair compared with the generic SF-36 ^(40)^. More studies should evaluate the effect of TENS on the quality of life using the Carolinas Comfort Scale.

No serious adverse events of TENS following inguinal hernia repair were observed in this study (moderate certainty of the evidence). Our finding that adverse events were mild, primarily involving erythema and itching at the electrode site, corresponds to safety assessments conducted by professional organizations ^(41)^. A previous systematic review of TENS for acute and chronic pain has obtained similar results ^(33)^. This supports the notion that TENS is generally a safe intervention for pain management, with only minor adverse effects that can be easily managed. Patients with electronic implants, such as cardiac pacemakers or implantable cardioverter defibrillators, are contraindicated for TENS. In the present review, it is noteworthy that the use of TENS is indicated for patients following inguinal hernia repair. This is because TENS is generally contraindicated to “patients with abdominal or inguinal hernias” prior to surgery. TENS should be used only in patients deemed appropriate by the surgeon, considering their specific condition and potential risks.

The exact mechanism of TENS on postoperative pain relief following inguinal hernia repair is unclear. One possible mechanism is that TENS selectively activates low-threshold somatosensory peripheral afferents, which has been demonstrated to decrease the activity and excitability of sensitized and nonsensitized central nociceptive transmitter cells ^(42), (43)^. Considering that the main cause of postoperative pain following inguinal hernia repair is damage to the pubic groin, iliopsoas, and femoropopliteal nerves, this may explain why local anesthesia was particularly effective in the present study. Furthermore, this effect may not last longer than the duration of the stimulation ^(42), (43)^, consistent with the finding that repetitive TENS was more effective than single TENS in the present study. The physiological intent of administering conventional TENS is to selectively activate nonnociceptive low-threshold afferent nerve fibers (Aβ fibers) in the skin, which are believed to inhibit the transmission of nociceptive information at the level of the spinal cord ^(44)^. The physiological purpose of acupuncture-like-TENS is to increase the activity of small afferent nerve fibers (Aδ) in the muscle, leading to muscle contractions that are thought to activate the descending pain suppression pathway ^(45)^. In this study, no significant differences were observed between conventional TENS and acupuncture-like-TENS. High-frequency TENS has been demonstrated to influence the pharmacological effects on the central nervous system in animal studies ^(46)^. In the present study, high-frequency TENS was more effective than low-frequency TENS, supporting the notion that higher frequencies may have a more significant impact on pain relief.

This study had several limitations. First, it included only 11 RCTs, and the sample size was small. However, we employed a rigorous methodology based on the PRISMA statement ^(14)^. Second, the included studies used different doses and types of TENS; however, we investigated subgroup analyses of the TENS doses, types, and frequency. Third, we could not evaluate chronic pain, but one RCT found no occurrence of chronic pain in either the placebo or TENS group ^(30)^. A previous Cochrane review on chronic pain did not provide conclusive evidence to determine whether TENS is harmful or beneficial for chronic pain control. This highlights the need for further high-quality research to better understand the efficacy of TENS in managing chronic pain and its potential impact on various aspects of patients’ lives.

Nonetheless, this review had several strengths. First, the protocol was registered with PROSPERO (CRD42022353932) and published in OSF (https://osf.io/) to improve transparency. Second, we searched nine electronic databases and two trial registries and carefully and rigorously designed the screening, extraction, and scoring processes according to the Cochrane handbook ^(17)^. Third, we evaluated and summarized the outcomes of interest with the certainty of the evidence using the GRADE approach ^(18)^.

In conclusion, this systematic review showed that TENS likely reduces pain up to POD 2 following inguinal hernia repair without increasing adverse events. Healthcare providers can choose to use TENS to reduce postoperative pain after inguinal hernia repair. Further large-scale trials with preregistered protocols are warranted to confirm our findings.

## Article Information

### Conflicts of Interest

None

### Acknowledgement

We thank Dr. Yuki Kataoka and Dr. Akihiro Shiroshita for providing us with the AMED and PsycINFO search data, respectively.

### Author Contributions

Study concept: JW; study design: JW; statistical analyses: JW; interpretation of data: JW; manuscript preparation: JW; manuscript editing: NI, FK, AM, and NS; manuscript review: NI, FK, AM, and NS; and literature screening: JW, NI, FK, and AM. All authors approved the final version and agreed to be accountable for the accuracy and integrity of the work.

### Approval by Institutional Review Board (IRB)

Not applicable.

## Supplement

Supplementary Supplementary 1: Electronic database search strategy
Supplementary 2: Trial registry search strategy
Supplementary 3: Risk of bias for the eligibility studies

Supplementary Figures Supplementary Figure 1: Forest plot of pain on postoperative day (POD) 1 by A) surgical approach (anterior approach vs. laparoscopy), B) transcutaneous electrical nerve stimulation types (conventional transcutaneous electrical nerve stimulation type vs. acupuncture-like transcutaneous electrical nerve stimulation types), C) anesthesia types (general vs. spinal vs. local), D) dose of transcutaneous electrical nerve stimulation (single vs. twice vs. repetitive), E) frequency of transcutaneous electrical nerve stimulation (high vs. low)
Supplementary Figure 2: Funnel plot of pain on POD 1
Supplementary Figure 3: Forest plot of the use of rescue analgesics by A) anesthesia types (general vs. spinal vs. local), B) dose of transcutaneous electrical nerve stimulation (single vs. repetitive)
Supplementary Figure 4: Forest plot of pain on POD 0 by A) transcutaneous electrical nerve stimulation types (conventional transcutaneous electrical nerve stimulation type vs. acupuncture-like transcutaneous electrical nerve stimulation types), B) anesthesia types (general vs. spinal vs. local), C) dose of transcutaneous electrical nerve stimulation (single vs. twice vs. repetitive), D) frequency of transcutaneous electrical nerve stimulation (high vs. low)
Supplementary Figure 5: Forest plot of pain on POD 2 by A) transcutaneous electrical nerve stimulation types (conventional transcutaneous electrical nerve stimulation type vs. acupuncture-like transcutaneous electrical nerve stimulation types), B) anesthesia types (general vs. spinal vs. local), C) dose of transcutaneous electrical nerve stimulation (single vs. twice vs. repetitive)
Supplementary Figure 6: Forest plot of pain on A) POD 1, B) POD 0, and C) POD 2, excluding studies with incomplete data
