# Supplementary material for: Efficacy and Safety of Transcutaneous Electrical Nerve Stimulation in Patients Undergoing Inguinal Hernia Repair: A Systematic Review and Meta-analysis
Source: JMA J. 2023 Sep 13;6(4):371–80. doi: 10.31662/jmaj.2023-0056 (PMC10628323; doi:10.31662/jmaj.2023-0056)
Supplement: Supplementary — 1: Electronic database search strategy Supplementary 2: Trial registry search strategy Supplementary 3: Risk of bias for the eligibility studies [file 2433-3298-6-4-371-s001.pdf]

Supplementary 1: Search strategy of the electronic databases

CENTRAL search strategy

#1. MeSH descriptor: [Hernia, Inguinal] explode all trees

#2. (Inguina* OR groin*):ti,ab,kw (Word variations have been searched)

#3. herni*:ti,ab,kw (Word variations have been searched)

#4. #2 AND #3

#5. #1 OR #4

#6. MeSH descriptor: [Transcutaneous electric nerve stimulation] explode all trees

#7. (TENS OR TNS OR ENS OR "Transcutaneous electric nerve stimulation" OR "transcutaneous nerve stimulation" OR "electric nerve stimulation" OR "electrostimulation" NEXT therap* OR "electro-stimulation" NEXT therap* OR electroanalgesi*):ti,ab,kw (Word variations have been searched)

#8. #6 OR #5

#9. #5 AND #8

(([mh "Hernia, Inguinal"]) OR ((Inguina*:ti,ab OR groin*:ti,ab) AND herni*:ti,ab)) AND ([mh "TRANSCUTANEOUS ELECTRIC NERVE STIMULATION"] OR TENS:ti,ab OR TNS:ti,ab OR ENS:ti,ab OR "Transcutaneous electric nerve

stimulation":ti,ab OR "transcutaneous nerve stimulation":ti,ab OR "electric nerve stimulation":ti,ab OR ("electrostimulation" NEXT therap*):ti,ab OR ("electro-stimulation" NEXT therap*):ti,ab OR electroanalgesi*:ti,ab)

MEDLINE (via PubMed) search strategy

#1. "Hernia, Inguinal"[Mesh]

#2. (Inguina*[tiab] OR groin*[tiab]) AND herni*[tiab]

#3. #1 OR #2

#4. "Transcutaneous Electric Nerve Stimulation"[MeSH]

#5. TENS[tiab] OR TNS[tiab] OR ENS[tiab] OR “Transcutaneous electric nerve stimulation”[tiab] or “transcutaneous nerve stimulation”[tiab] OR “electric nerve stimulation”[tiab] or “electrostimulation therap*”[tiab] or “electro-stimulation therap*”[tiab] or electroanalgesi*[tiab]

#6. #4 OR #5

#7. #3 AND #6

(("Hernia, Inguinal"[Mesh]) OR ((Inguina*[tiab] OR groin*[tiab]) AND herni*[tiab]) AND ("TRANSCUTANEOUS ELECTRIC NERVE STIMULATION"[Mesh] OR

TENS[tiab] OR TNS[tiab] OR ENS[tiab] OR "Transcutaneous electric nerve stimulation"[tiab] OR "transcutaneous nerve stimulation"[tiab] OR "electric nerve stimulation"[tiab] OR "electrostimulation therap*"[tiab] OR "electro-stimulation therap*"[tiab] OR electroanalgesi*[tiab])

EMBASE (via ProQuest Dialog) search strategy

S1 EMB.EXACT.EXPLODE("inguinal hernia")

S2 ab(Inguinal OR groin) OR ti(Inguinal OR groin)

S3 ab(hernia) OR ti(hernia)

S4 S2 AND S3

S5 S1 OR S4

S6 EMB.EXACT.EXPLODE("transcutaneous electric nerve stimulation")

S7 ab(TENS OR TNS OR ENS OR "Transcutaneous electric nerve stimulation" OR "transcutaneous nerve stimulation" OR "electric nerve stimulation" OR "electrostimulation therapy" OR "electro-stimulation therap*" OR electroanalgesia) OR ti(TENS OR TNS OR ENS OR "Transcutaneous electric nerve stimulation" OR "transcutaneous nerve stimulation" OR "electric nerve stimulation" OR "electrostimulation therapy" OR "electro-stimulation therap*" OR electroanalgesia)

S8 S6 OR S7

S9 S5 AND S8

CINAHAL

(((MH "Hernia, Inguinal"+)) OR (((TI Inguina* OR AB Inguina*) OR (TI groin* OR AB groin*)) AND (TI herni* OR AB herni*)) AND ((MH "TRANSCUTANEOUS ELECTRIC NERVE STIMULATION"+) OR (TI TENS OR AB TENS) OR (TI TNS OR AB TNS) OR (TI ENS OR AB ENS) OR (TI "Transcutaneous electric nerve stimulation" OR AB "Transcutaneous electric nerve stimulation") OR (TI "transcutaneous nerve stimulation" OR AB "transcutaneous nerve stimulation") OR (TI "electric nerve stimulation" OR AB "electric nerve stimulation") OR (TI "electrostimulation therap*" OR AB "electrostimulation therap*") OR (TI "electro-stimulation therap*" OR AB "electro-stimulation therap*") OR (TI electroanalgesi* OR AB electroanalgesi*)))

AMED

1. exp "Hernia, Inguinal"

2. (“Inguinal” or “groin”).ti,ab.

3. (“hernia“).ti,ab.
4. 2 and 3
5. 1 or 4
6. exp Transcutaneous Electric Nerve Stimulation/
7. ("TENS" or "TNS" or "ENS").ti,ab.
8. ("transcutaneous electric nerve stimulation" or "transcutaneous electrical nerve stimulation" or "transcutaneous nerve stimulation").tw.
9. ("electric nerve stimulation" or "electrical nerve stimulation" or "electrostimulation therap$" or "electro-stimulation therap$").tw.
10. ("electric nerve therap$" or "electrical nerve therap$" or electroanalgesi$).tw.
11. ("transcutaneous electric stimulation" or "transcutaneous electrical stimulation").tw.
12. TES.ti,ab.
13. or/6-12
14. 5 and 13

PsycINFO

((exp "Hernia, Inguinal"/) OR ((Inguina*.ti,ab. OR groin*.ti,ab.) AND herni*.ti,ab.)

AND (exp "TRANSCUTANEOUS ELECTRIC NERVE STIMULATION"/ OR

TENS.ti,ab. OR TNS.ti,ab. OR ENS.ti,ab. OR "Transcutaneous electric nerve stimulation".ti,ab. OR "transcutaneous nerve stimulation".ti,ab. OR "electric nerve stimulation".ti,ab. OR "electrostimulation therap*".ti,ab. OR "electro-stimulation therap*".ti,ab. OR electroanalgesi*.ti,ab.))

Web of science

(("Hernia, Inguinal") OR ((Inguina* OR groin*) AND herni*) AND ("TRANSCUTANEOUS ELECTRIC NERVE STIMULATION" OR TENS OR TNS OR ENS OR "Transcutaneous electric nerve stimulation" OR "transcutaneous nerve stimulation" OR "electric nerve stimulation" OR "electrostimulation therap*" OR "electro-stimulation therap*" OR electroanalgesi*))

Supplementary 2: Clinical trial registries

ClinicalTrials.gov. search strategy

Condition or disease: (Inguinal AND hernia)

Intervention/treatment: (TENS OR TNS OR ENS OR “Transcutaneous electric nerve stimulation” or “transcutaneous nerve stimulation” OR “electric nerve stimulation” OR “electrostimulation therapy” or “electro-stimulation therapy” or electroanalgesia)

ICTRP search strategy

((Inguinal OR groin) AND hernia) AND (TENS OR TNS OR ENS OR "Transcutaneous electric nerve stimulation" or "transcutaneous nerve stimulation" OR "electric nerve stimulation" OR "electrostimulation therapy" or "electro-stimulation therapy" or electroanalgesia)

Supplementary 3: Risk of bias for the eligibility studies

The use of rescue analgesics

Authors [ref no.]	Risk of bias 2 tool assessment					
	Bias arising from the randomization process	Bias due to deviations from intended interventions	Bias due to missing outcome data	Bias in measurement of the outcome	Bias in selection of the reported results	Overall risk of bias
Gilbert [19]	Some concerns	Low	Low	Low	Some concerns	Some concerns
Smedley [20]	Some concerns	Low	Low	Low	Some concerns	Some concerns
Ahmed [22]	Low	Low	Some concerns	Low	High	High
Eidy [25]	Low	Low	Low	Low	Low	Low
Gorganchian [26]	Some concerns	Low	Low	Low	Some concerns	Some concerns

Quality of life

Authors [ref no.]	Risk of bias 2 tool assessment					
	Bias arising from the randomization process	Bias due to deviations from intended interventions	Bias due to missing outcome data	Bias in measurement of the outcome	Bias in selection of the reported results	Overall risk of bias
Parseliunas [28]	Low	Low	Low	Low	Low	Low

Adverse events

Authors [ref no.]	Risk of bias 2 tool assessment					
	Bias arising from the randomization process	Bias due to deviations from intended interventions	Bias due to missing outcome data	Bias in measurement of the outcome	Bias in selection of the reported results	Overall risk of bias
DeSantana [21]	Low	Low	Low	Low	Some concerns	Some concerns
Dias [23]	Low	Low	Low	Low	Some concerns	Some concerns
Szmit [29]	Low	Low	Low	Low	Low	Low
