# Supplementary material for: Efficacy and Safety of Transcutaneous Electrical Nerve Stimulation in Patients Undergoing Inguinal Hernia Repair: A Systematic Review and Meta-analysis
Source: JMA J. 2023 Sep 13;6(4):371–80. doi: 10.31662/jmaj.2023-0056 (PMC10628323; doi:10.31662/jmaj.2023-0056)

Supplementary figure 1

A

Study or Subgroup	TENS Mean	TENS SD	TENS Total	Control Mean	Control SD	Control Total	Weight	Std. Mean Difference IV, Random, 95% CI
**1.1.1 Anterior approach**								
Gilbert 1986	3.26	0.7	20	3.37	0.69	20	11.4%	-0.16 [-0.78, 0.47]
Smedley 1988	5.5	2.3	34	5.1	1.7	28	11.7%	0.19 [-0.31, 0.69]
DeSantana 2008	0	0	20	3.4	2.2	20		Not estimable
Ahmed 2010	4.93	0.7	30	6.61	0.69	30	11.2%	-2.39 [-3.06, -1.71]
Dias 2010	2.26	0.7	16	3.1	0.69	17	11.0%	-1.18 [-1.93, -0.43]
Dalamagka 2015	0.63	0.9898	36	1.1	1.48	18	11.5%	-0.39 [-0.97, 0.18]
Eidy 2016	3.51	1.5	33	3.57	1.5	33	11.7%	-0.04 [-0.52, 0.44]
Gorganchian 2016	0.62	0.7	12	5.1	2.22	12	9.7%	-2.63 [-3.77, -1.49]
Yilmaz 2018	1	0.5	26	4	0.74	26	9.9%	-4.68 [-5.76, -3.60]
Parseliunas 2021	0.75	0.84	40	1.2	0.84	40	11.8%	-0.53 [-0.98, -0.08]
**Subtotal (95% CI)**			**267**			**244**	**100.0%**	**-1.23 [-2.01, -0.45]**
Heterogeneity: Tau² = 1.29; Chi² = 112.69, df = 8 (P < 0.00001); I² = 93%								
Test for overall effect: Z = 3.08 (P = 0.002)								
**1.1.2 Laparoscopy**								
Szmit 2021	1.3	1	24	2.9	1.5	24	100.0%	-1.23 [-1.86, -0.61]
**Subtotal (95% CI)**			**24**			**24**	**100.0%**	**-1.23 [-1.86, -0.61]**
Heterogeneity: Not applicable								
Test for overall effect: Z = 3.89 (P < 0.0001)								

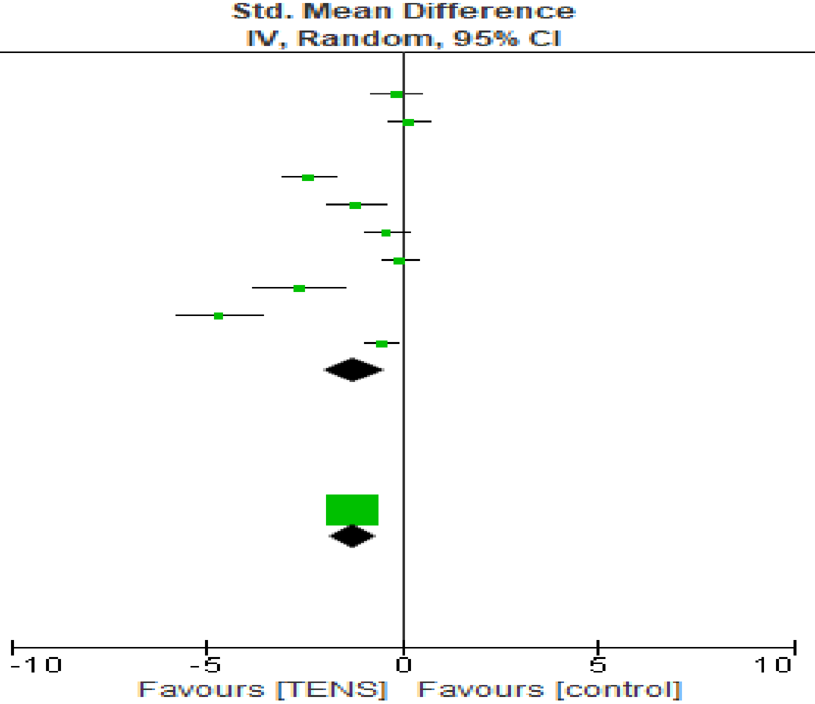

Test for subgroup differences: Chi² = 0.00, df = 1 (P = 0.99), I² = 0%

B

Study or Subgroup	TENS Mean	TENS SD	TENS Total	Control Mean	Control SD	Control Total	Weight	Std. Mean Difference IV, Random, 95% CI
**1.2.1 Conventional TENS**								
Gilbert 1986	3.26	0.7	20	3.37	0.69	20	12.8%	-0.16 [-0.78, 0.47]
Smedley 1988	5.5	2.3	34	5.1	1.7	28	13.1%	0.19 [-0.31, 0.69]
DeSantana 2008	0	0	20	3.4	2.2	20		Not estimable
Ahmed 2010	4.93	0.7	30	6.61	0.69	30	12.6%	-2.39 [-3.06, -1.71]
Eidy 2016	3.51	1.5	33	3.57	1.5	33	13.1%	-0.04 [-0.52, 0.44]
Gorganchian 2016	0.62	0.7	12	5.1	2.22	12	11.1%	-2.63 [-3.77, -1.49]
Yilmaz 2018	1	0.5	26	4	0.74	26	11.3%	-4.68 [-5.76, -3.60]
Szmit 2021	1.3	1	24	2.9	1.5	24	12.8%	-1.23 [-1.86, -0.61]
Parseliunas 2021	0.75	0.84	40	1.2	0.84	40	13.2%	-0.53 [-0.98, -0.08]
**Subtotal (95% CI)**			**239**			**233**	**100.0%**	**-1.35 [-2.23, -0.47]**
Heterogeneity: Tau² = 1.46; Chi² = 112.54, df = 7 (P < 0.00001); I² = 94%								
Test for overall effect: Z = 3.02 (P = 0.003)								
**1.2.2 AL-TENS**								
Dias 2010	2.26	0.7	16	3.1	0.69	17	45.1%	-1.18 [-1.93, -0.43]
Dalamagka 2015	0.63	0.9898	36	1.1	1.48	18	54.9%	-0.39 [-0.97, 0.18]
**Subtotal (95% CI)**			**52**			**35**	**100.0%**	**-0.75 [-1.51, 0.02]**
Heterogeneity: Tau² = 0.19; Chi² = 2.67, df = 1 (P = 0.10); I² = 63%								
Test for overall effect: Z = 1.92 (P = 0.06)								

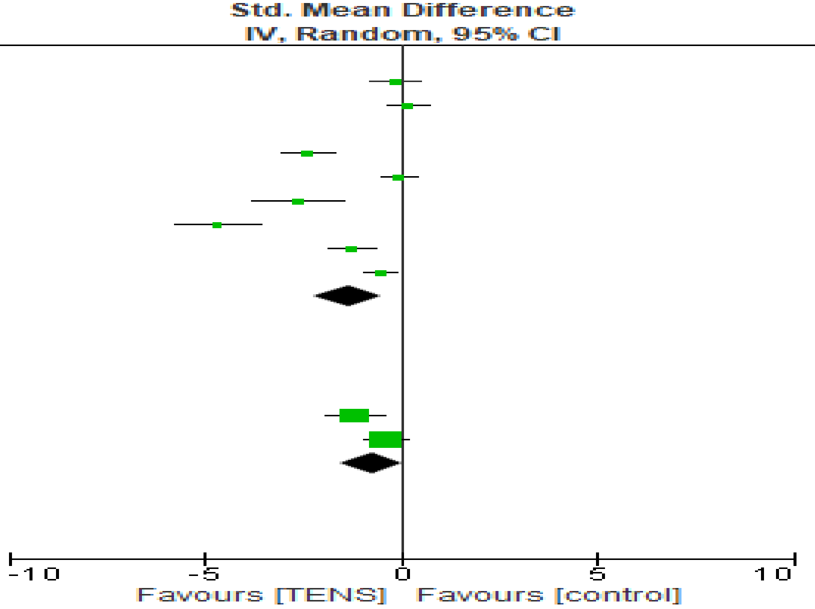

Test for subgroup differences: Chi² = 1.03, df = 1 (P = 0.31), I² = 2.5%

Supplementary figure 1

C

Study or Subgroup	TENS Mean	TENS SD	TENS Total	Control Mean	Control SD	Control Total	Weight	Std. Mean Difference IV, Random, 95% CI
**1.3.1 General**								
Smedley 1988	5.5	2.3	34	5.1	1.7	28	34.5%	0.19 [-0.31, 0.69]
Dalamagka 2015	0.63	0.9898	36	1.1	1.48	18	33.2%	-0.39 [-0.97, 0.18]
Szmit 2021	1.3	1	24	2.9	1.5	24	32.2%	-1.23 [-1.86, -0.61]
**Subtotal (95% CI)**			**94**			**70**	**100.0%**	**-0.46 [-1.27, 0.34]**
Heterogeneity: Tau^2 = 0.42; Chi^2 = 12.27, df = 2 (P = 0.002); I^2 = 84%								
Test for overall effect: Z = 1.13 (P = 0.26)								
**1.3.2 Spinal**								
Gilbert 1986	3.26	0.7	20	3.37	0.69	20	23.1%	-0.16 [-0.78, 0.47]
DeSantana 2008	0	0	20	3.4	2.2	20		Not estimable
Eidy 2016	3.51	1.5	33	3.57	1.5	33	35.9%	-0.04 [-0.52, 0.44]
Parseliunas 2021	0.75	0.84	40	1.2	0.84	40	41.0%	-0.53 [-0.98, -0.08]
**Subtotal (95% CI)**			**113**			**113**	**100.0%**	**-0.27 [-0.58, 0.05]**
Heterogeneity: Tau^2 = 0.01; Chi^2 = 2.32, df = 2 (P = 0.31); I^2 = 14%								
Test for overall effect: Z = 1.67 (P = 0.09)								
**1.3.3 Local**								
Ahmed 2010	4.93	0.7	30	6.61	0.69	30	26.5%	-2.39 [-3.06, -1.71]
Dias 2010	2.26	0.7	16	3.1	0.69	17	26.1%	-1.18 [-1.93, -0.43]
Gorganchian 2016	0.62	0.7	12	5.1	2.22	12	23.5%	-2.63 [-3.77, -1.49]
Yilmaz 2018	1	0.5	26	4	0.74	26	23.9%	-4.68 [-5.76, -3.60]
**Subtotal (95% CI)**			**84**			**85**	**100.0%**	**-2.68 [-4.00, -1.35]**
Heterogeneity: Tau^2 = 1.61; Chi^2 = 27.45, df = 3 (P < 0.00001); I^2 = 89%								
Test for overall effect: Z = 3.96 (P < 0.0001)								

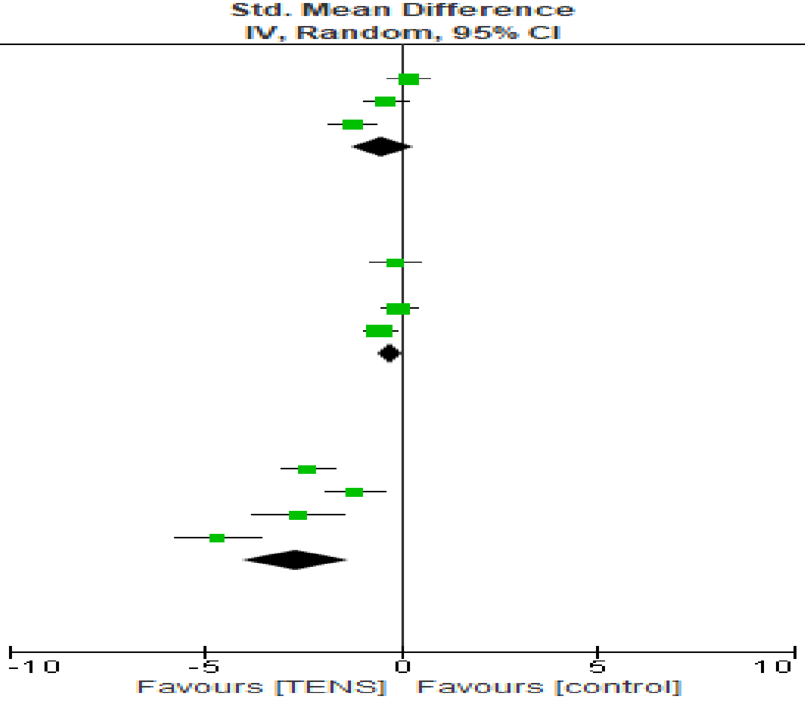

Test for subgroup differences: Chi^2 = 12.03, df = 2 (P = 0.002), I^2 = 83.4%

D

Study or Subgroup	TENS Mean	TENS SD	TENS Total	Control Mean	Control SD	Control Total	Weight	Std. Mean Difference IV, Random, 95% CI
**1.4.1 Single**								
Gilbert 1986	3.26	0.7	20	3.37	0.69	20	23.9%	-0.16 [-0.78, 0.47]
Smedley 1988	5.5	2.3	34	5.1	1.7	28	36.6%	0.19 [-0.31, 0.69]
Eidy 2016	3.51	1.5	33	3.57	1.5	33	39.5%	-0.04 [-0.52, 0.44]
**Subtotal (95% CI)**			**87**			**81**	**100.0%**	**0.02 [-0.29, 0.32]**
Heterogeneity: Tau^2 = 0.00; Chi^2 = 0.82, df = 2 (P = 0.66); I^2 = 0%								
Test for overall effect: Z = 0.12 (P = 0.91)								
**1.4.2 Twice**								
DeSantana 2008	0	0	20	3.4	2.2	20		Not estimable
Dias 2010	2.26	0.7	16	3.1	0.69	17	100.0%	-1.18 [-1.93, -0.43]
**Subtotal (95% CI)**			**36**			**37**	**100.0%**	**-1.18 [-1.93, -0.43]**
Heterogeneity: Not applicable								
Test for overall effect: Z = 3.09 (P = 0.002)								
**1.4.3 Repetitive**								
Ahmed 2010	4.93	0.7	30	6.61	0.69	30	17.1%	-2.39 [-3.06, -1.71]
Dalamagka 2015	0.63	0.9898	36	1.1	1.48	18	17.4%	-0.39 [-0.97, 0.18]
Gorganchian 2016	0.62	0.7	12	5.1	2.22	12	15.1%	-2.63 [-3.77, -1.49]
Yilmaz 2018	1	0.5	26	4	0.74	26	15.4%	-4.68 [-5.76, -3.60]
Szmit 2021	1.3	1	24	2.9	1.5	24	17.2%	-1.23 [-1.86, -0.61]
Parseliunas 2021	0.75	0.84	40	1.2	0.84	40	17.8%	-0.53 [-0.98, -0.08]
**Subtotal (95% CI)**			**168**			**150**	**100.0%**	**-1.90 [-2.96, -0.84]**
Heterogeneity: Tau^2 = 1.59; Chi^2 = 73.76, df = 5 (P < 0.00001); I^2 = 93%								
Test for overall effect: Z = 3.52 (P = 0.0004)								

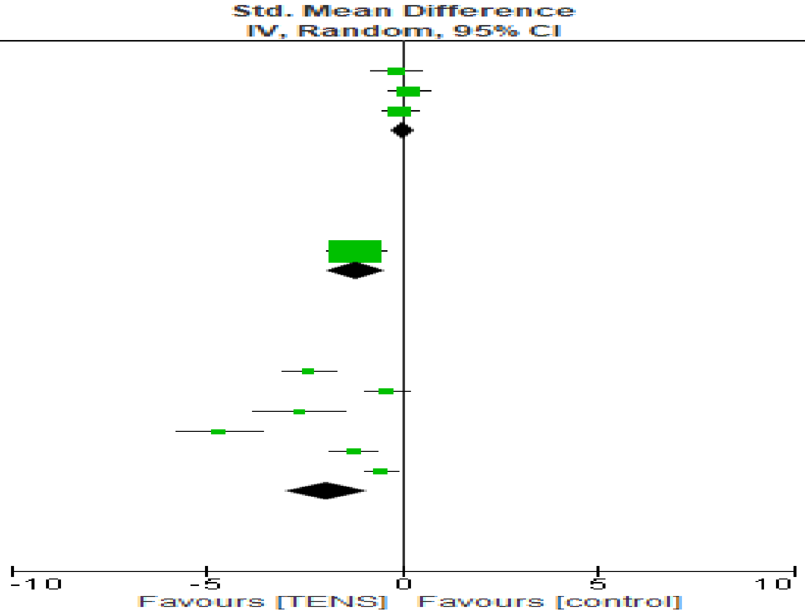

Test for subgroup differences: Chi^2 = 18.26, df = 2 (P = 0.0001), I^2 = 89.0%

Supplementary figure 1

E

Study or Subgroup	TENS Mean	TENS SD	TENS Total	Control Mean	Control SD	Control Total	Weight	Std. Mean Difference IV, Random, 95% CI	Year
**1.5.1 High frequency**									
Gilbert 1986	3.26	0.7	20	3.37	0.69	20	12.9%	-0.16 [-0.78, 0.47]	1986
Smedley 1988	5.5	2.3	34	5.1	1.7	28	13.2%	0.19 [-0.31, 0.69]	1988
DeSantana 2008	0	0	20	3.4	2.2	20		Not estimable	2008
Ahmed 2010	4.93	0.7	30	6.61	0.69	30	12.7%	-2.39 [-3.06, -1.71]	2010
Dias 2010	2.26	0.7	16	3.1	0.69	17	12.5%	-1.18 [-1.93, -0.43]	2010
Gorganchian 2016	0.62	0.7	12	5.1	2.22	12	11.2%	-2.63 [-3.77, -1.49]	2016
Yilmaz 2018	1	0.5	26	4	0.74	26	11.4%	-4.68 [-5.76, -3.60]	2018
Szmit 2021	1.3	1	24	2.9	1.5	24	12.9%	-1.23 [-1.86, -0.61]	2021
Parseliunas 2021	0.75	0.84	40	1.2	0.84	40	13.3%	-0.53 [-0.98, -0.08]	2021
**Subtotal (95% CI)**			**222**			**217**	**100.0%**	**-1.50 [-2.39, -0.61]**	
Heterogeneity: Tau² = 1.50; Chi² = 102.30, df = 7 (P < 0.00001); I² = 93%									
Test for overall effect: Z = 3.32 (P = 0.0009)									
**1.5.2 Low frequency**									
Dalamagka 2015	0.63	0.9898	36	1.1	1.48	18	100.0%	-0.39 [-0.97, 0.18]	2015
**Subtotal (95% CI)**			**36**			**18**	**100.0%**	**-0.39 [-0.97, 0.18]**	
Heterogeneity: Not applicable									
Test for overall effect: Z = 1.36 (P = 0.18)									

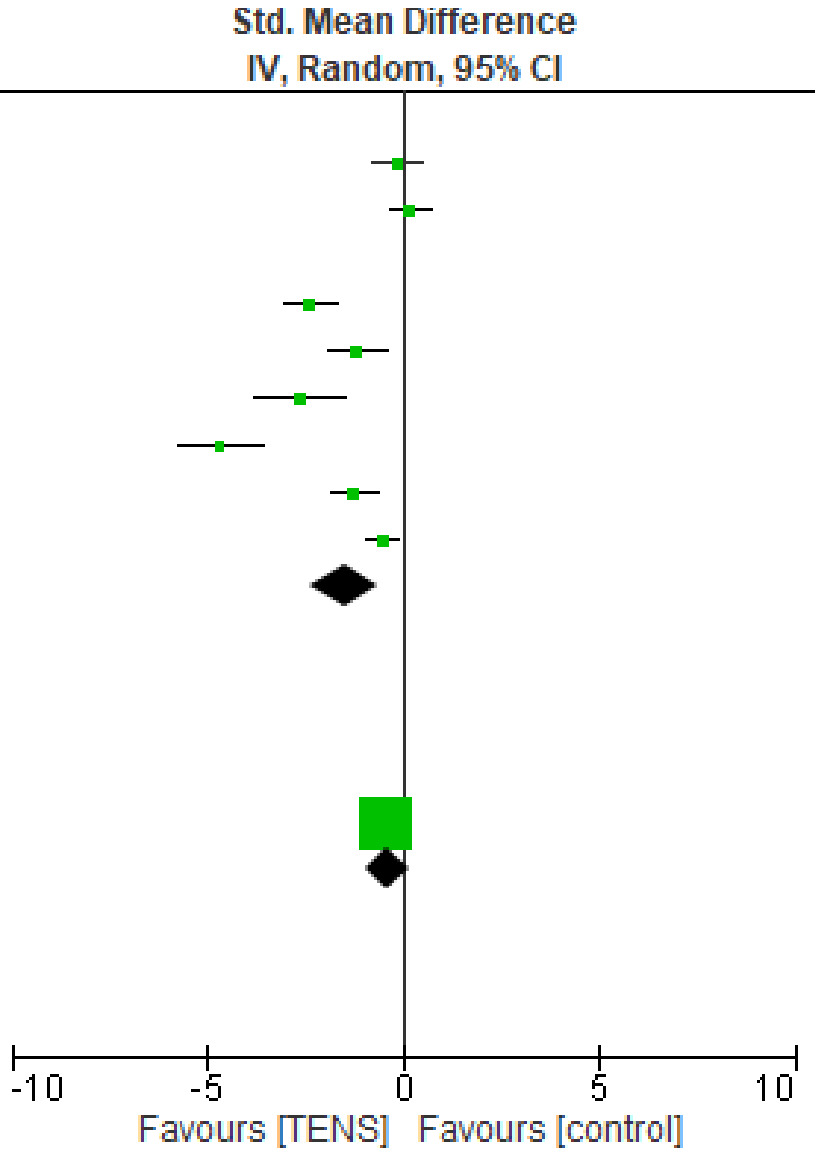

Test for subgroup differences: Chi² = 4.23, df = 1 (P = 0.04), I² = 76.4%

Supplementary figure 2

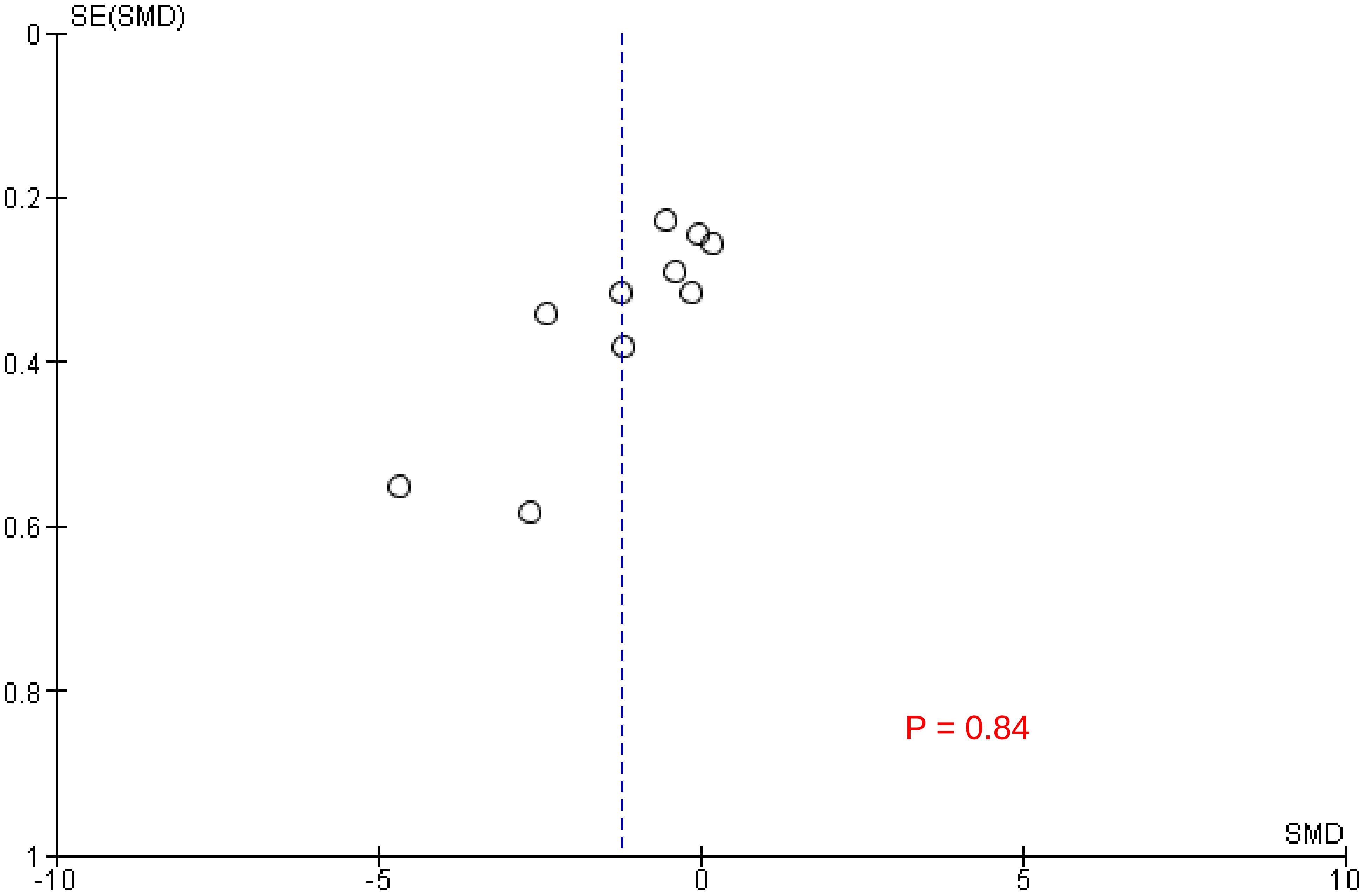

Supplementary figure 3

A

Study or Subgroup	TENS Events	TENS Total	Control Events	Control Total	Weight	Risk Ratio M-H, Random, 95% CI
**2.1.1 General**						
Smedley 1988	21	34	15	28	100.0%	1.15 [0.75, 1.78]
**Subtotal (95% CI)**		**34**		**28**	**100.0%**	**1.15 [0.75, 1.78]**
Total events	21		15			
Heterogeneity: Not applicable						
Test for overall effect: Z = 0.64 (P = 0.52)						
**2.1.2 Spinal**						
Gilbert 1986	8	20	8	20	53.5%	1.00 [0.47, 2.14]
Eidy 2016	5	33	13	33	46.5%	0.38 [0.15, 0.96]
**Subtotal (95% CI)**		**53**		**53**	**100.0%**	**0.64 [0.25, 1.66]**
Total events	13		21			
Heterogeneity: $Tau^2 = 0.29$; $Chi^2 = 2.58$, df = 1 (P = 0.11); $I^2 = 61\%$						
Test for overall effect: Z = 0.92 (P = 0.36)						
**2.1.3 Local**						
Ahmed 2010	19	30	27	30	61.1%	0.70 [0.52, 0.95]
Gorganchian 2016	0	12	7	12	38.9%	0.07 [0.00, 1.05]
**Subtotal (95% CI)**		**42**		**42**	**100.0%**	**0.28 [0.02, 4.79]**
Total events	19		34			
Heterogeneity: $Tau^2 = 3.40$; $Chi^2 = 4.40$, df = 1 (P = 0.04); $I^2 = 77\%$						
Test for overall effect: Z = 0.88 (P = 0.38)						

Test for subgroup differences: $Chi^2 = 2.01$, df = 2 (P = 0.37), $I^2 = 0.7\%$

Risk Ratio M-H, Random, 95% CI

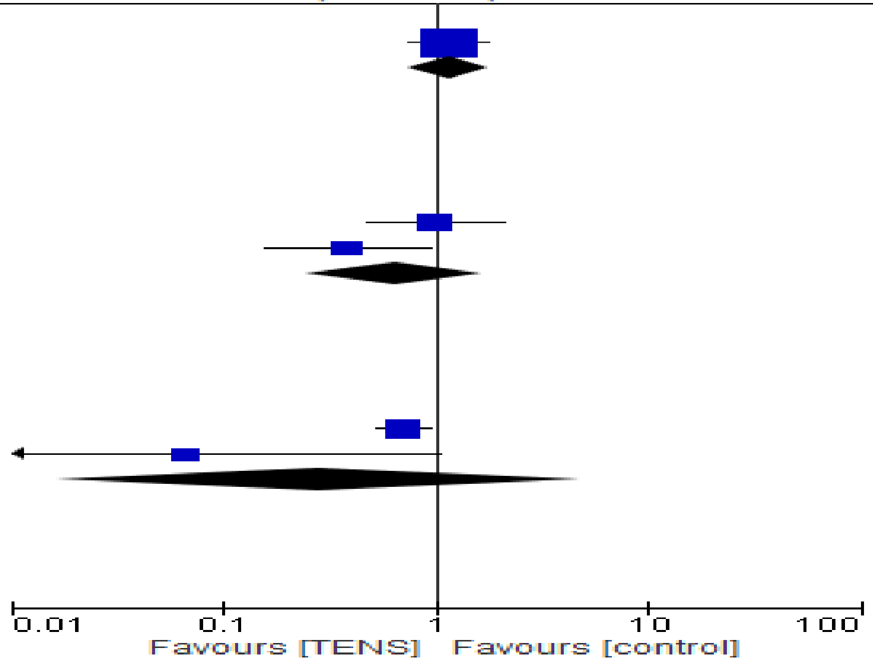

B

Study or Subgroup	TENS Events	TENS Total	Control Events	Control Total	Weight	Risk Ratio M-H, Random, 95% CI
**2.2.1 Single**						
Gilbert 1986	8	20	8	20	30.5%	1.00 [0.47, 2.14]
Smedley 1988	21	34	15	28	44.1%	1.15 [0.75, 1.78]
Eidy 2016	5	33	13	33	25.4%	0.38 [0.15, 0.96]
**Subtotal (95% CI)**		**87**		**81**	**100.0%**	**0.84 [0.45, 1.55]**
Total events	34		36			
Heterogeneity: $Tau^2 = 0.18$; $Chi^2 = 4.94$, df = 2 (P = 0.08); $I^2 = 60\%$						
Test for overall effect: Z = 0.57 (P = 0.57)						
**2.2.2 Repetitive**						
Ahmed 2010	19	30	27	30	61.1%	0.70 [0.52, 0.95]
Gorganchian 2016	0	12	7	12	38.9%	0.07 [0.00, 1.05]
**Subtotal (95% CI)**		**42**		**42**	**100.0%**	**0.28 [0.02, 4.79]**
Total events	19		34			
Heterogeneity: $Tau^2 = 3.40$; $Chi^2 = 4.40$, df = 1 (P = 0.04); $I^2 = 77\%$						
Test for overall effect: Z = 0.88 (P = 0.38)						

Test for subgroup differences: $Chi^2 = 0.54$, df = 1 (P = 0.46), $I^2 = 0\%$

Risk Ratio M-H, Random, 95% CI

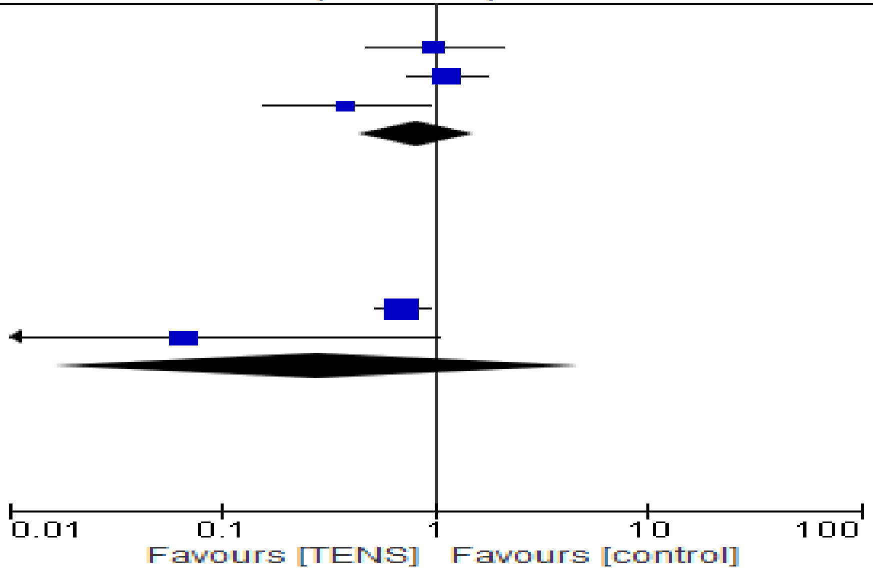

Supplementary figure 4

A

Study or Subgroup	TENS Mean	TENS SD	TENS Total	Control Mean	Control SD	Control Total	Weight	Std. Mean Difference IV, Random, 95% CI
**3.1.1 Conventional TENS**								
Smedley 1988	6.8	1.3	34	7.3	1.6	28	26.1%	-0.34 [-0.85, 0.16]
DeSantana 2008	1.7	1.2	20	5.9	3.3	20	25.5%	-1.66 [-2.39, -0.93]
Eidy 2016	4.15	1.25	33	4.27	1.35	33	26.2%	-0.09 [-0.57, 0.39]
Yılmaz 2018	2	0.46	26	5	0.37	26	22.2%	-7.08 [-8.59, -5.56]
**Subtotal (95% CI)**			**113**			**107**	**100.0%**	**-2.11 [-3.85, -0.36]**
Heterogeneity: Tau² = 2.97; Chi² = 82.59, df = 3 (P < 0.00001); I² = 96%								
Test for overall effect: Z = 2.37 (P = 0.02)								
**3.1.2 AL-TENS**								
Dias 2010	1.4	1.4	16	2.6	2.4	17	50.5%	-0.59 [-1.29, 0.11]
Dalamagka 2015	2.35	0.5837	36	4.43	0.4	18	49.5%	-3.86 [-4.81, -2.92]
**Subtotal (95% CI)**			**52**			**35**	**100.0%**	**-2.21 [-5.42, 0.99]**
Heterogeneity: Tau² = 5.17; Chi² = 29.75, df = 1 (P < 0.00001); I² = 97%								
Test for overall effect: Z = 1.35 (P = 0.18)								

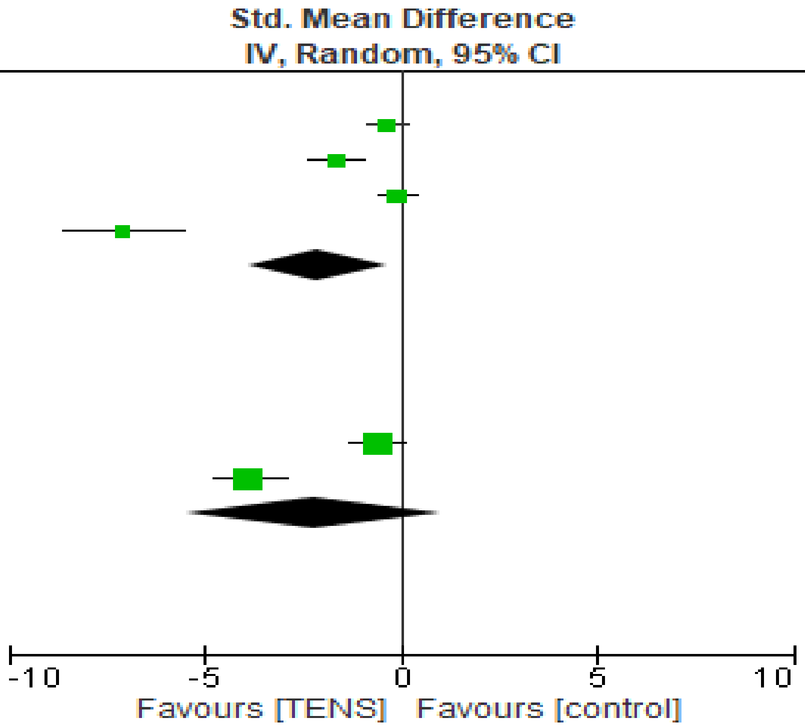

Test for subgroup differences: Chi² = 0.00, df = 1 (P = 0.96), I² = 0%

B

Study or Subgroup	TENS Mean	TENS SD	TENS Total	Control Mean	Control SD	Control Total	Weight	Std. Mean Difference IV, Random, 95% CI
**3.2.1 General**								
Smedley 1988	6.8	1.3	34	7.3	1.6	28	34.1%	-0.34 [-0.85, 0.16]
Dalamagka 2015	2.35	0.5837	36	4.43	0.4	18	31.8%	-3.86 [-4.81, -2.92]
Eidy 2016	4.15	1.25	33	4.27	1.35	33	34.1%	-0.09 [-0.57, 0.39]
**Subtotal (95% CI)**			**103**			**79**	**100.0%**	**-1.38 [-3.13, 0.38]**
Heterogeneity: Tau² = 2.28; Chi² = 51.00, df = 2 (P < 0.00001); I² = 96%								
Test for overall effect: Z = 1.54 (P = 0.12)								
**3.2.2 Spinal**								
DeSantana 2008	1.7	1.2	20	5.9	3.3	20	100.0%	-1.66 [-2.39, -0.93]
**Subtotal (95% CI)**			**20**			**20**	**100.0%**	**-1.66 [-2.39, -0.93]**
Heterogeneity: Not applicable								
Test for overall effect: Z = 4.46 (P < 0.00001)								
**3.2.3 Local**								
Dias 2010	1.4	1.4	16	2.6	2.4	17	50.6%	-0.59 [-1.29, 0.11]
Yılmaz 2018	2	0.46	26	5	0.37	26	49.4%	-7.08 [-8.59, -5.56]
**Subtotal (95% CI)**			**42**			**43**	**100.0%**	**-3.80 [-10.16, 2.56]**
Heterogeneity: Tau² = 20.68; Chi² = 58.00, df = 1 (P < 0.00001); I² = 98%								
Test for overall effect: Z = 1.17 (P = 0.24)								

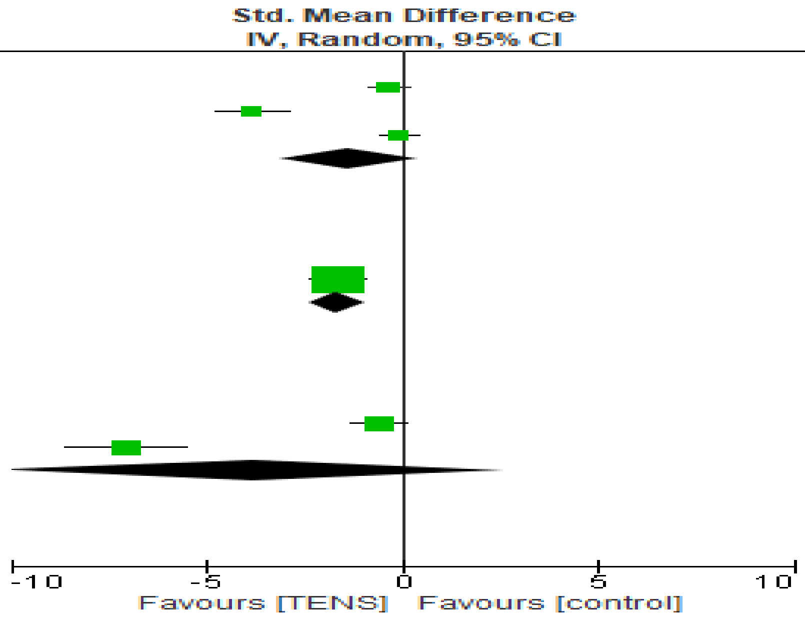

Test for subgroup differences: Chi² = 0.53, df = 2 (P = 0.77), I² = 0%

Supplementary figure 4

C

Study or Subgroup	TENS Mean	TENS SD	TENS Total	Control Mean	Control SD	Control Total	Weight	Std. Mean Difference IV, Random, 95% CI
**3.3.1 Single**								
Smedley 1988	6.8	1.3	34	7.3	1.6	28	47.8%	-0.34 [-0.85, 0.16]
Eidy 2016	4.15	1.25	33	4.27	1.35	33	52.2%	-0.09 [-0.57, 0.39]
**Subtotal (95% CI)**			**67**			**61**	**100.0%**	**-0.21 [-0.56, 0.14]**
Heterogeneity: Tau² = 0.00; Chi² = 0.50, df = 1 (P = 0.48); I² = 0%								
Test for overall effect: Z = 1.19 (P = 0.23)								
**3.3.2 Twice**								
DeSantana 2008	1.7	1.2	20	5.9	3.3	20	49.5%	-1.66 [-2.39, -0.93]
Dias 2010	1.4	1.4	16	2.6	2.4	17	50.5%	-0.59 [-1.29, 0.11]
**Subtotal (95% CI)**			**36**			**37**	**100.0%**	**-1.12 [-2.16, -0.07]**
Heterogeneity: Tau² = 0.44; Chi² = 4.29, df = 1 (P = 0.04); I² = 77%								
Test for overall effect: Z = 2.10 (P = 0.04)								
**3.3.3 Repetitive**								
Dalamagka 2015	2.35	0.5837	36	4.43	0.4	18	51.8%	-3.86 [-4.81, -2.92]
Yilmaz 2018	2	0.46	26	5	0.37	26	48.2%	-7.08 [-8.59, -5.56]
**Subtotal (95% CI)**			**62**			**44**	**100.0%**	**-5.41 [-8.56, -2.26]**
Heterogeneity: Tau² = 4.76; Chi² = 12.45, df = 1 (P = 0.0004); I² = 92%								
Test for overall effect: Z = 3.37 (P = 0.0008)								

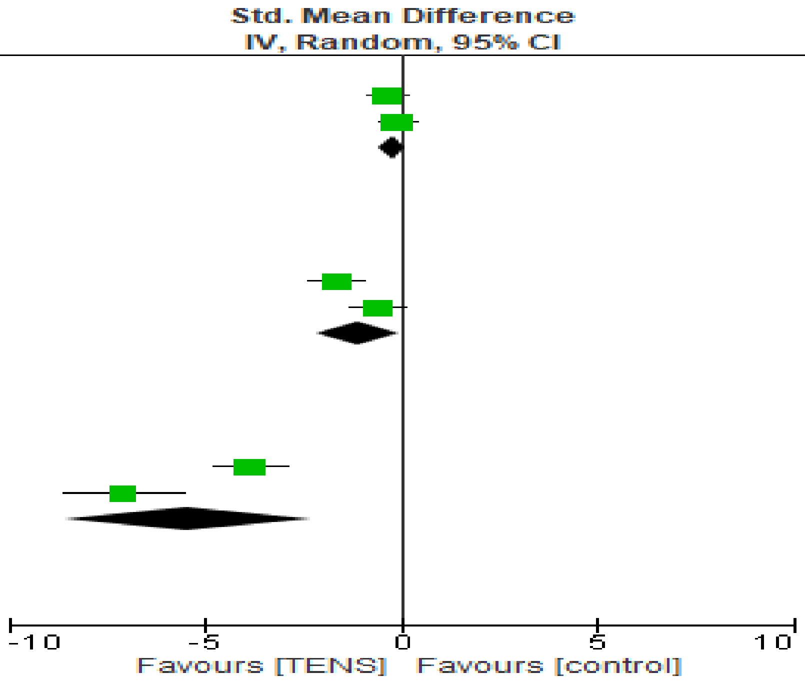

Test for subgroup differences: Chi² = 12.62, df = 2 (P = 0.002), I² = 84.1%

D

Study or Subgroup	TENS Mean	TENS SD	TENS Total	Control Mean	Control SD	Control Total	Weight	Std. Mean Difference IV, Random, 95% CI
**3.4.1 High frequency**								
Smedley 1988	6.8	1.3	34	7.3	1.6	28	26.1%	-0.34 [-0.85, 0.16]
DeSantana 2008	1.7	1.2	20	5.9	3.3	20	25.6%	-1.66 [-2.39, -0.93]
Dias 2010	1.4	1.4	16	2.6	2.4	17	25.7%	-0.59 [-1.29, 0.11]
Yilmaz 2018	2	0.46	26	5	0.37	26	22.6%	-7.08 [-8.59, -5.56]
**Subtotal (95% CI)**			**96**			**91**	**100.0%**	**-2.26 [-4.11, -0.42]**
Heterogeneity: Tau² = 3.31; Chi² = 72.66, df = 3 (P < 0.00001); I² = 96%								
Test for overall effect: Z = 2.41 (P = 0.02)								
**3.4.2 Low frequency**								
Dalamagka 2015	2.35	0.5837	36	4.43	0.4	18	100.0%	-3.86 [-4.81, -2.92]
**Subtotal (95% CI)**			**36**			**18**	**100.0%**	**-3.86 [-4.81, -2.92]**
Heterogeneity: Not applicable								
Test for overall effect: Z = 8.01 (P < 0.00001)								

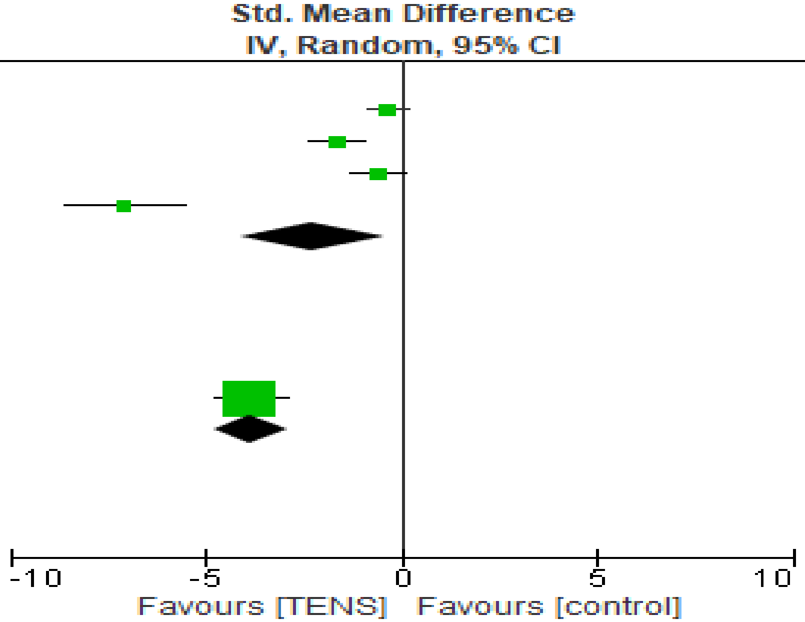

Test for subgroup differences: Chi² = 2.29, df = 1 (P = 0.13), I² = 56.4%

Supplementary figure 5

A

Study or Subgroup	TENS Mean	TENS SD	TENS Total	Control Mean	Control SD	Control Total	Weight	Std. Mean Difference IV, Random, 95% CI
**4.1.1 Conventional TENS**								
Gilbert 1986	2.59	0.57	20	2.13	0.54	20	24.7%	0.81 [0.16, 1.46]
Smedley 1988	4.56	1.84	34	5.06	1.78	28	25.4%	-0.27 [-0.77, 0.23]
Ahmed 2010	4.05	0.57	30	5.6	0.54	30	24.3%	-2.76 [-3.47, -2.04]
Parseliunas 2021	0.48	0.4	40	0.94	0.6	40	25.6%	-0.89 [-1.35, -0.43]
**Subtotal (95% CI)**			**124**			**118**	**100.0%**	**-0.77 [-1.99, 0.46]**
Heterogeneity: Tau² = 1.47; Chi² = 55.80, df = 3 (P < 0.00001); I² = 95%								
Test for overall effect: Z = 1.23 (P = 0.22)								
**4.1.2 AL-TENS**								
Dias 2010	1.58	0.57	16	2.63	0.54	17	100.0%	-1.85 [-2.68, -1.02]
**Subtotal (95% CI)**			**16**			**17**	**100.0%**	**-1.85 [-2.68, -1.02]**
Heterogeneity: Not applicable								
Test for overall effect: Z = 4.35 (P < 0.0001)								

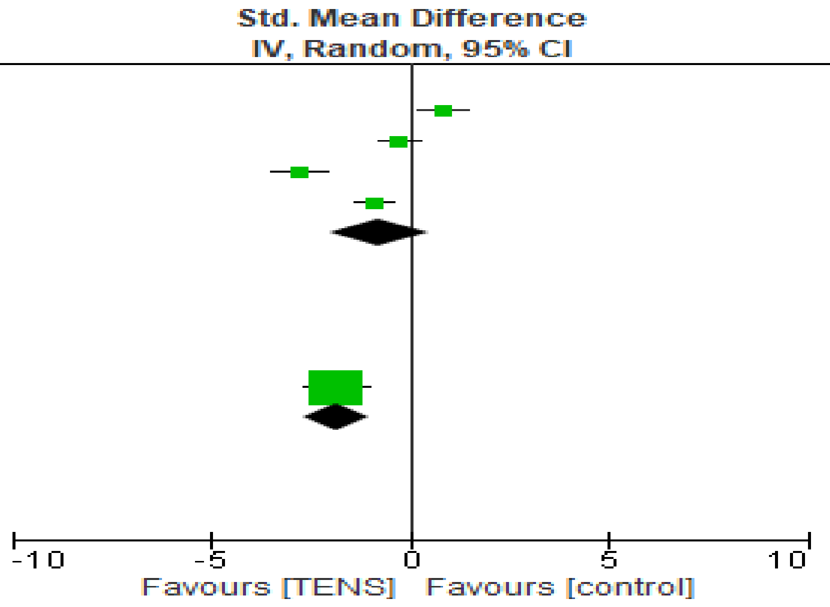

Test for subgroup differences: Chi² = 2.04, df = 1 (P = 0.15), I² = 51.1%

B

Study or Subgroup	TENS Mean	TENS SD	TENS Total	Control Mean	Control SD	Control Total	Weight	Std. Mean Difference IV, Random, 95% CI	Year
**4.2.1 General**									
Smedley 1988	4.56	1.84	34	5.06	1.78	28	100.0%	-0.27 [-0.77, 0.23]	1988
**Subtotal (95% CI)**			**34**			**28**	**100.0%**	**-0.27 [-0.77, 0.23]**	
Heterogeneity: Not applicable									
Test for overall effect: Z = 1.06 (P = 0.29)									
**4.2.2 Spinal**									
Gilbert 1986	2.59	0.57	20	2.13	0.54	20	49.1%	0.81 [0.16, 1.46]	1986
Parseliunas 2021	0.48	0.4	40	0.94	0.6	40	50.9%	-0.89 [-1.35, -0.43]	2021
**Subtotal (95% CI)**			**60**			**60**	**100.0%**	**-0.06 [-1.73, 1.61]**	
Heterogeneity: Tau² = 1.37; Chi² = 17.69, df = 1 (P < 0.0001); I² = 94%									
Test for overall effect: Z = 0.07 (P = 0.95)									
**4.2.3 Local**									
Ahmed 2010	4.05	0.57	30	5.6	0.54	30	52.8%	-2.76 [-3.47, -2.04]	2010
Dias 2010	1.58	0.57	16	2.63	0.54	17	47.2%	-1.85 [-2.68, -1.02]	2010
**Subtotal (95% CI)**			**46**			**47**	**100.0%**	**-2.33 [-3.22, -1.44]**	
Heterogeneity: Tau² = 0.26; Chi² = 2.63, df = 1 (P = 0.11); I² = 62%									
Test for overall effect: Z = 5.13 (P < 0.00001)									

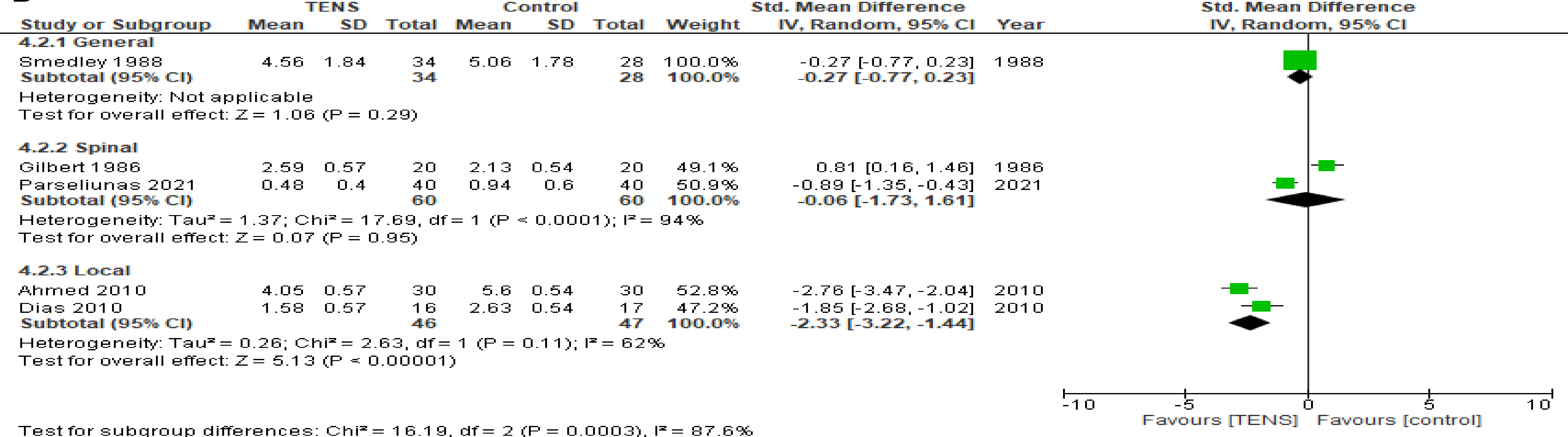

Test for subgroup differences: Chi² = 16.19, df = 2 (P = 0.0003), I² = 87.6%

Supplementary figure 5

C

Study or Subgroup	TENS Mean	TENS SD	TENS Total	Control Mean	Control SD	Control Total	Weight	Std. Mean Difference IV, Random, 95% CI	Year
**4.3.1 Single**									
Gilbert 1986	2.59	0.57	20	2.13	0.54	20	48.2%	0.81 [0.16, 1.46]	1986
Smedley 1988	4.56	1.84	34	5.06	1.78	28	51.8%	-0.27 [-0.77, 0.23]	1988
**Subtotal (95% CI)**			**54**			**48**	**100.0%**	**0.25 [-0.81, 1.31]**	
Heterogeneity: $Tau^2 = 0.50$; $Chi^2 = 6.72$, df = 1 (P = 0.010); $I^2 = 85\%$									
Test for overall effect: Z = 0.46 (P = 0.64)									
**4.3.2 Twice**									
Dias 2010	1.58	0.57	16	2.63	0.54	17	100.0%	-1.85 [-2.68, -1.02]	2010
**Subtotal (95% CI)**			**16**			**17**	**100.0%**	**-1.85 [-2.68, -1.02]**	
Heterogeneity: Not applicable									
Test for overall effect: Z = 4.35 (P < 0.0001)									
**4.3.3 Repetitive**									
Ahmed 2010	4.05	0.57	30	5.6	0.54	30	48.9%	-2.76 [-3.47, -2.04]	2010
Parseliunas 2021	0.48	0.4	40	0.94	0.6	40	51.1%	-0.89 [-1.35, -0.43]	2021
**Subtotal (95% CI)**			**70**			**70**	**100.0%**	**-1.80 [-3.63, 0.02]**	
Heterogeneity: $Tau^2 = 1.64$; $Chi^2 = 18.28$, df = 1 (P < 0.0001); $I^2 = 95\%$									
Test for overall effect: Z = 1.94 (P = 0.05)									

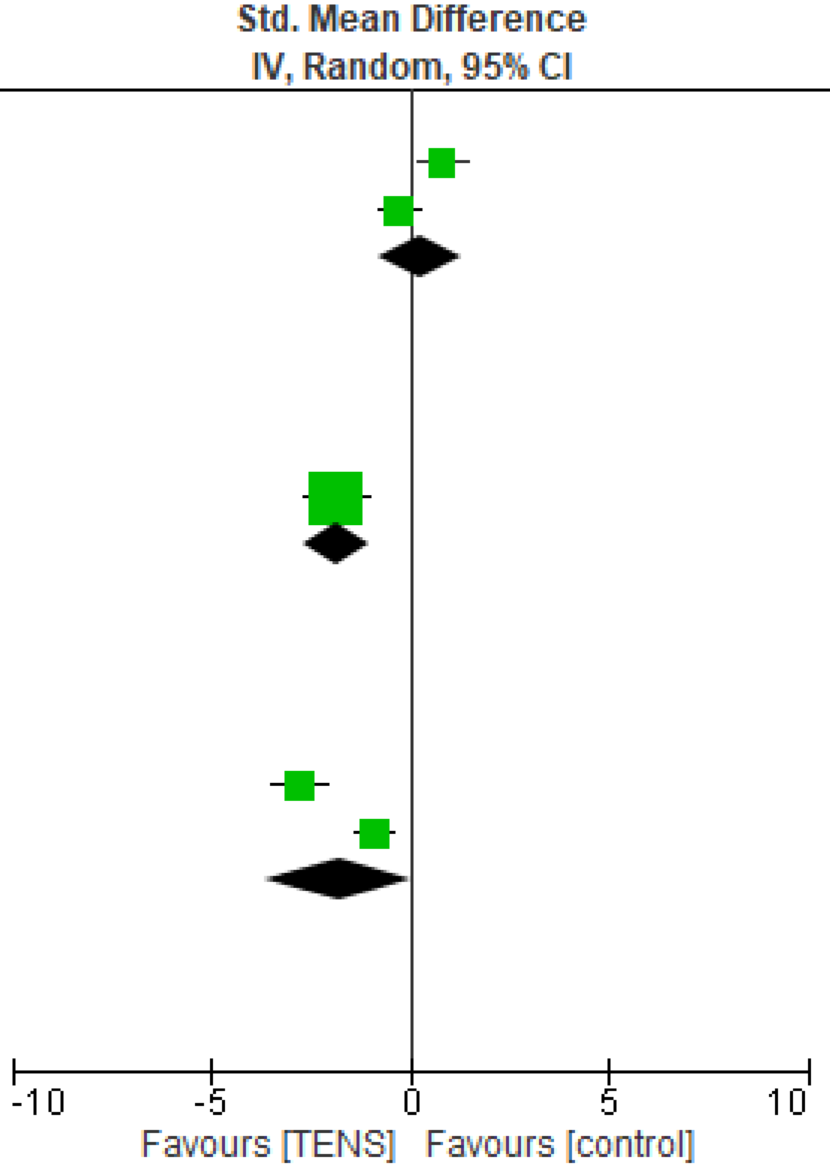

Test for subgroup differences: $Chi^2 = 9.86$, df = 2 (P = 0.007), $I^2 = 79.7\%$

Supplementary figure 6

A

Study or Subgroup	TENS Mean	TENS SD	TENS Total	Control Mean	Control SD	Control Total	Weight	Std. Mean Difference IV, Random, 95% CI
Gilbert 1986	3.26	0.7	20	3.37	0.69	20	12.6%	-0.16 [-0.78, 0.47]
Smedley 1988	5.5	2.3	34	5.1	1.7	28	13.2%	0.19 [-0.31, 0.69]
DeSantana 2008	0	0	20	3.4	2.2	20		Not estimable
Ahmed 2010	4.93	0.7	30	6.61	0.69	30	12.3%	-2.39 [-3.06, -1.71]
Dalamagka 2015	0.63	0.9898	36	1.1	1.48	18	12.9%	-0.39 [-0.97, 0.18]
Eidy 2016	3.51	1.5	33	3.57	1.5	33	13.3%	-0.04 [-0.52, 0.44]
Gorganchian 2016	0.62	0.7	12	5.1	2.22	12	9.6%	-2.63 [-3.77, -1.49]
Szmit 2021	1.3	1	24	2.9	1.5	24	12.6%	-1.23 [-1.86, -0.61]
Parseliunas 2021	0.75	0.84	40	1.2	0.84	40	13.5%	-0.53 [-0.98, -0.08]
**Total (95% CI)**			**249**			**225**	**100.0%**	**-0.82 [-1.43, -0.21]**

Heterogeneity: $Tau^2 = 0.67$; $Chi^2 = 60.59$, df = 7 ($P < 0.00001$); $I^2 = 88\%$
Test for overall effect: Z = 2.65 (P = 0.008)

Std. Mean Difference IV, Random, 95% CI

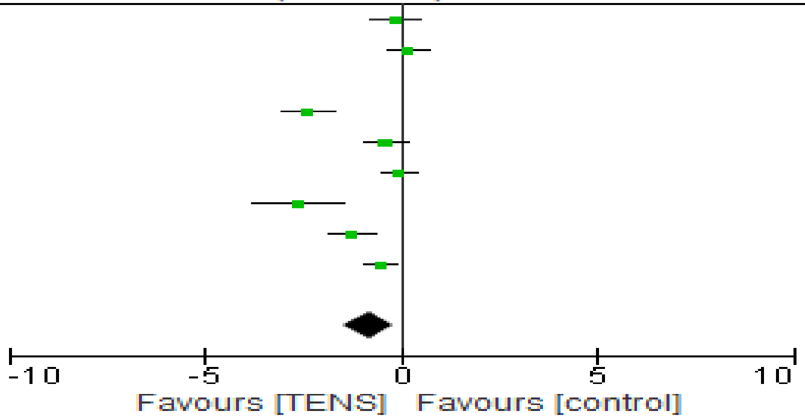

B

Study or Subgroup	TENS Mean	TENS SD	TENS Total	Control Mean	Control SD	Control Total	Weight	Std. Mean Difference IV, Random, 95% CI
Smedley 1988	6.8	1.3	34	7.3	1.6	28	25.7%	-0.34 [-0.85, 0.16]
DeSantana 2008	1.7	1.2	20	5.9	3.3	20	24.8%	-1.66 [-2.39, -0.93]
Dalamagka 2015	2.35	0.5837	36	4.43	0.4	18	23.6%	-3.86 [-4.81, -2.92]
Eidy 2016	4.15	1.25	33	4.27	1.35	33	25.8%	-0.09 [-0.57, 0.39]
**Total (95% CI)**			**123**			**99**	**100.0%**	**-1.44 [-2.80, -0.07]**

Heterogeneity: $Tau^2 = 1.81$; $Chi^2 = 57.13$, df = 3 ($P < 0.00001$); $I^2 = 95\%$
Test for overall effect: Z = 2.07 (P = 0.04)

Std. Mean Difference IV, Random, 95% CI

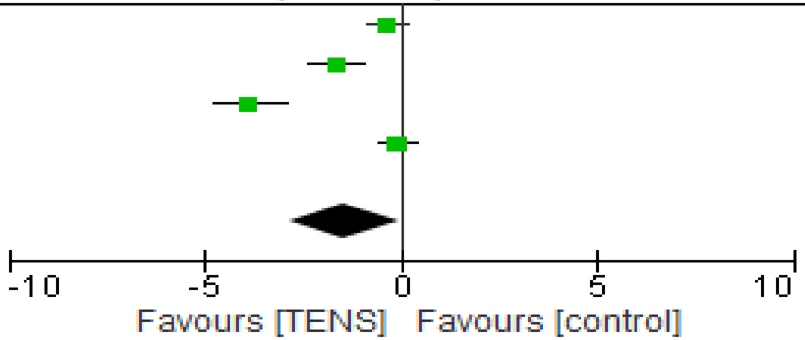

C

Study or Subgroup	TENS Mean	TENS SD	TENS Total	Control Mean	Control SD	Control Total	Weight	Std. Mean Difference IV, Random, 95% CI
Gilbert 1986	2.59	0.57	20	2.13	0.54	20	24.7%	0.81 [0.16, 1.46]
Smedley 1988	4.56	1.84	34	5.06	1.78	28	25.4%	-0.27 [-0.77, 0.23]
Ahmed 2010	4.05	0.57	30	5.6	0.54	30	24.3%	-2.76 [-3.47, -2.04]
Parseliunas 2021	0.48	0.4	40	0.94	0.6	40	25.6%	-0.89 [-1.35, -0.43]
**Total (95% CI)**			**124**			**118**	**100.0%**	**-0.77 [-1.99, 0.46]**

Heterogeneity: $Tau^2 = 1.47$; $Chi^2 = 55.80$, df = 3 ($P < 0.00001$); $I^2 = 95\%$
Test for overall effect: Z = 1.23 (P = 0.22)

Std. Mean Difference IV, Random, 95% CI

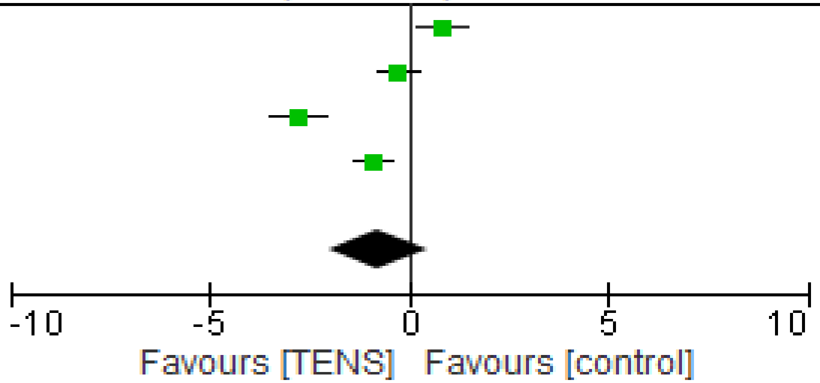

Supplement: Supplementary Figures — Supplementary Figure 1: Forest plot of pain on postoperative day (POD) 1 by A) surgical approach (anterior approach vs. laparoscopy), B) transcutaneous electrical nerve stimulation types (conventional transcutaneous electrical nerve stimulation type vs. acupuncture-like transcutaneous electrical nerve stimulation types), C) anesthesia types (general vs. spinal vs. local), D) dose of transcutaneous electrical nerve stimulation (single vs. twice vs. repetitive), E) frequency of transcutaneous electrical nerve stimulation (high vs. low) Supplementary Figure 2: Funnel plot of pain on POD 1 Supplementary Figure 3: Forest plot of the use of rescue analgesics by A) anesthesia types (general vs. spinal vs. local), B) dose of transcutaneous electrical nerve stimulation (single vs. repetitive) Supplementary Figure 4: Forest plot of pain on POD 0 by A) transcutaneous electrical nerve stimulation types (conventional transcutaneous electrical nerve stimulation type vs. acupuncture-like transcutaneous electrical nerve stimulation types), B) anesthesia types (general vs. spinal vs. local), C) dose of transcutaneous electrical nerve stimulation (single vs. twice vs. repetitive), D) frequency of transcutaneous electrical nerve stimulation (high vs. low) Supplementary Figure 5: Forest plot of pain on POD 2 by A) transcutaneous electrical nerve stimulation types (conventional transcutaneous electrical nerve stimulation type vs. acupuncture-like transcutaneous electrical nerve stimulation types), B) anesthesia types (general vs. spinal vs. local), C) dose of transcutaneous electrical nerve stimulation (single vs. twice vs. repetitive) Supplementary Figure 6: Forest plot of pain on A) POD 1, B) POD 0, and C) POD 2, excluding studies with incomplete data [file 2433-3298-6-4-371-s002.pdf]
